# Supplementary material for: Assessing tobacco use in Swedish young adults from self-report and urinary cotinine: a validation study using the BAMSE birth cohort
Source: BMJ Open. 2023 Jul 12;13(7):e072582. doi: 10.1136/bmjopen-2023-072582 (PMC10347476; doi:10.1136/bmjopen-2023-072582)
Supplement: Supplementary data [file bmjopen-2023-072582supp001.pdf]

BMJ Publishing Group Limited (BMJ) disclaims all liability and responsibility arising from any reliance placed on this supplemental material which has been supplied by the author(s)

Assessing tobacco use in Swedish young adults from self-report and urinary cotinine: a validation study using the BAMSE birth cohort

Supplementary information

Anna Zettergren, Shanzina Iasmin Sompa, Lena Palmberg, Petter Ljungman, Göran Pershagen, Niklas Andersson, Christian H Lindh, Antonios Georgelis, Inger Kull, Erik Melén, Sandra Ekström, Anna Bergström

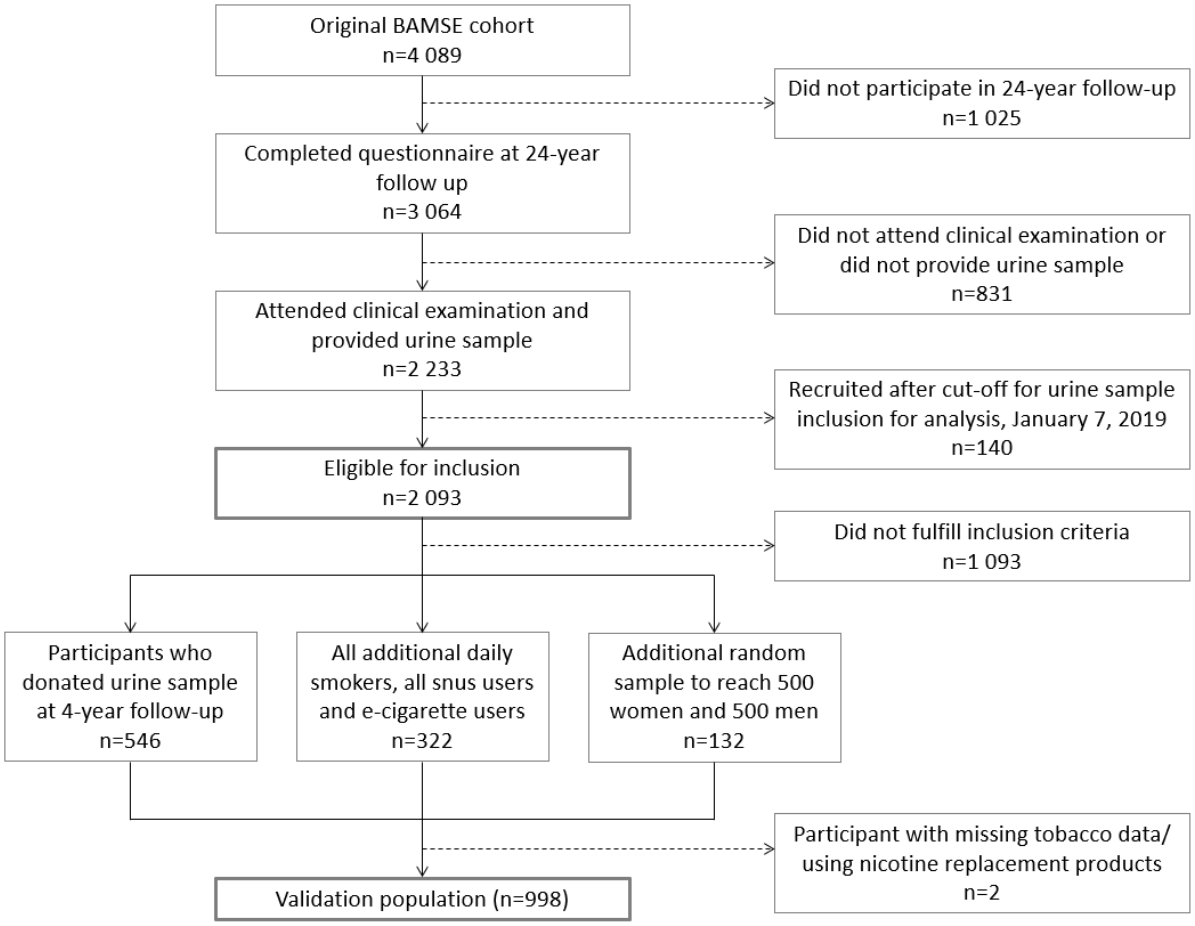

Figure S1. Overview of the selection process of the validation population for urine sample analysis of cotinine.

Variable definitions:

Age: Age in years at questionnaire, as calculated from date of birth.

Parental socioeconomic status: Household economic status at enrollment in the BAMSE study. Categorized as i) blue collar worker or ii) white collar worker, based on the highest employment category of the parents.

Any parent smoking at baseline: Any parent smoking at baseline of the BAMSE cohort. Based on self-reported questionnaire data from parents.

Education level: Participants highest completed education level at the time of the 24-year follow-up questionnaire. Categorized as i) elementary school or high school and ii) university or higher.

Occupation: Based on questionnaire data from 24-year follow-up. Categorized as i) student, ii) working or iii) other.

Body mass index (BMI): Calculated as kg/m^2 from weight and height measurements from the 24-year follow-up clinical examination.

Secondhand smoke exposure: Based on self-reported exposure from the 24-year follow-up questionnaire. Categorized as i) "yes" if daily exposed to indoor tobacco smoke (at home, work or other places), otherwise as ii) "no".

Asthma: Defined based on information from the 24-year follow-up questionnaire as "yes" if participants had a doctor's diagnosis of Asthma together with either symptoms of breathing difficulties in the last 12 months OR taking asthma medication in the past 12 months.

Table S1. Self-reported tobacco use in the validation population (n=998).

	All n=998	Women n=499	Men n=499	p-value[a] (women vs. men)
		n (%)		
**Cigarettes**				
Never	588 (59.0)	279 (56.0)	309 (61.9)	0.054
Former	135 (13.5)	60 (12.0)	75 (15.0)	0.165
Occasional	125 (12.5)	64 (12.8)	61 (12.2)	0.774
Daily	150 (15.0)	96 (19.2)	54 (10.8)	<0.0001
<10 cigarettes/day	*82 (55.4)*	*57 (60.0)*	*25 (47.2)*	*0.132*
≥10 cigarettes/day	*66 (44.6)*	*38 (40.0)*	*28 (52.8)*	
**Snus**				
Never	704 (70.5)	429 (86.0)	275 (55.1)	<0.0001
Former	37 (3.7)	5 (1.0)	32 (6.4)	<0.0001
Occasional	75 (7.5)	28 (5.6)	47 (9.4)	0.023
Daily	182 (18.2)	37 (7.4)	145 (29.1)	<0.0001
<4 boxes of snus/week	*99 (54.4)*	*27 (73.0)*	*72 (49.7)*	*0.011*
≥4 boxes of snus/week	*83 (45.6)*	*10 (27.0)*	*73 (50.3)*	
**E-cigarettes**				
Never	918 (92.0)	472 (94.6)	446 (89.4)	0.002
Occasional	71 (7.1)	24 (4.8)	47 (9.4)	0.005
Daily	9 (0.9)	3 (0.6)	6 (1.2)	0.315
**Waterpipe**				
Never	970 (97.4)	488 (97.8)	482 (97.0)	0.421
Occasional	25 (2.5)	10 (2.0)	15 (3.0)	0.306
Daily	1 (0.1)	1 (0.2)	0 (0)	0.318
Number of tobacco types used				
1	393 (77.4)	190 (84.1)	203 (72.0)	0.001
>1	115 (22.6)	36 (15.9)	79 (28.0)	
Secondhand smoke exposure				
Yes[1]	39 (4.0)	24 (4.9)	15 (3.1)	0.141

[a]: P-value from Pearson χ^2 test.
[b]: P-value from two-tailed t-test.
[c]: P-value from Wilcoxon rank-sum test
[1]: Defined as daily exposure to indoor tobacco smoke.

Table S2. Urinary cotinine levels (ng/ml) by tobacco use categories, adjusted for specific gravity.

	n	Median	Mean	SD	95th percentile	Min-max
**Cigarettes**						
Any cigarette smoking	275	1 465	2 089	2 176	6 215	0.1–14 399
Any cigarette smoking, mixed users excluded	*176*	*1 180*	*1 494*	*1 545*	*4 528*	*0.1–8 238*
Daily cigarette smoking	150	2 094	2 390	1 670	5 264	3.6–8 238
Daily cigarette smoking, mixed users excluded	*112*	*1 826*	*2 101*	*1 547*	*4 957*	*3.6–8 238*
Occasional cigarette smoking	125	422	1 728	2 621	7 449	0.1–14 399
Occasional cigarette smoking, mixed users excluded	*64*	*109*	*433*	*787*	*1 733*	*0.1–3 868*
**Snus**						
Any snus use	257	2 939	3 159	2 510	7 790	0.3–14 399
Any snus use, mixed users excluded	*174*	*2 566*	*2 834*	*2 368*	*7 381*	*0.3–10 350*
Daily snus use	182	3 599	3 950	2 346	8 051	1.7–14 399
Daily snus use, mixed users excluded	*119*	*3 449*	*3 726*	*2 125*	*7 859*	*1.7–10 350*
Occasional snus use	75	345	1 239	1 752	4 707	0.3–7 790
Occasional snus use, mixed users excluded	*55*	*161*	*903*	*1 500*	*4 088*	*0.3–7 790*
**E-cigarettes**						
Any e-cigarette smoking	80	874	2 159	2 697	8 359	0.3–9 999
Any e-cigarette smoking, mixed users excluded	*32*	*10.7*	*1 219*	*2 260*	*6 958*	*0.5–8 574*
Daily e-cigarette smoking	9	2 749	3 643	2 431	6 998	265–6 998
Daily e-cigarette smoking, mixed users excluded	*7*	*2 368*	*3 074*	*2 357*	*6 958*	*265–6 958*
Occasional e-cigarette smoking	71	527	1 971	2 686	8 574	0.3–9 999
Occasional e-cigarette smoking, mixed users excluded	*25*	*5.7*	*700*	*1 981*	*4 714*	*0.5–8 574*
**Waterpipe**						
Any waterpipe use	26	1 415	2 400	2 698	6 602	0.3–9 169
Any waterpipe use, mixed users excluded	*9*	*2.0*	*7.2*	*10.1*	*28.9*	*0.7–28.9*

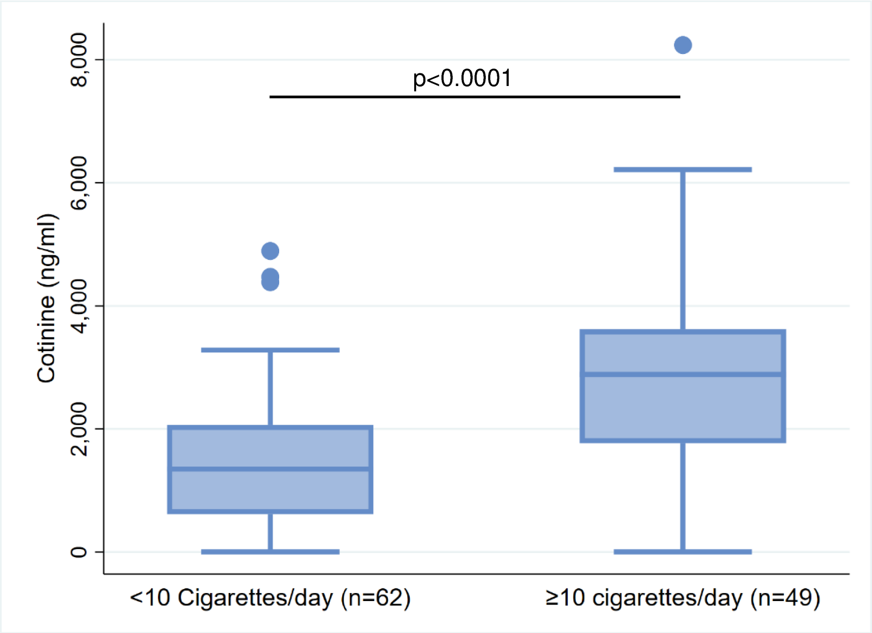

Figure S2. Urinary cotinine levels among daily cigarette smokers, by number of cigarettes per day. Participants with mixed tobacco use excluded.

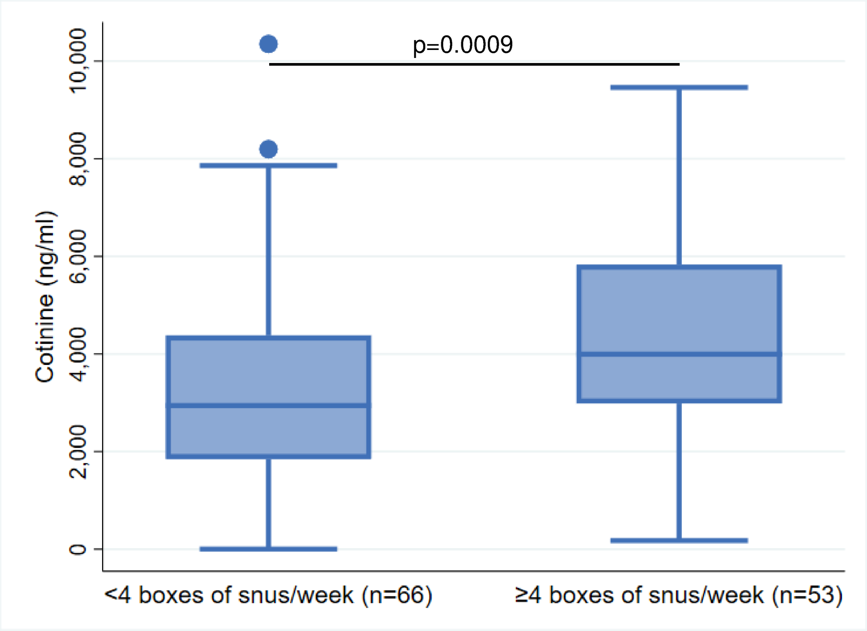

Figure S3. Urinary cotinine levels among daily snus users, by number of boxes snus per week. Participants with mixed tobacco use excluded.

Table S3. Self-reported tobacco use against urinary cotinine levels by type of tobacco and frequency of use, stratified by gender.

			Women	Men
Cigarettes	Daily	κ:	0.838	0.801
		Sensitivity:	83.3%	75.4%
		Specificity:	97.7%	99.0%
		PVV:	93.8%	96.3%
		NPV:	93.4%	92.2%
	Occasional	κ:	0.592	0.643
		Sensitivity:	69.5%	72.1%
		Specificity:	91.7%	92.2%
		PVV:	64.1%	72.1%
		NPV:	93.4%	92.2%
	Any	κ:	0.764	0.760
		Sensitivity:	87.9%	85.0%
		Specificity:	89.8%	91.3%
		PVV:	81.9%	83.5%
		NPV:	93.4%	92.2%
Snus	Daily	κ:	0.726	0.898
		Sensitivity:	65.4%	89.4%
		Specificity:	98.8%	99.5%
		PVV:	91.9%	99.3%
		NPV:	93.4%	92.2%
	Occasional	κ:	0.461	0.640
		Sensitivity:	47.1%	67.3%
		Specificity:	95.5%	94.3%
		PVV:	57.1%	74.5%
		NPV:	93.4%	92.2%
	Any	κ:	0.691	0.853
		Sensitivity:	73.5%	91.3%
		Specificity:	94.4%	93.9%
		PVV:	76.9%	93.2%
		NPV:	93.4%	92.2%
E-cigarette use	Any	κ:	0.418	0.658
		Sensitivity:	43.8%	70.2%
		Specificity:	95.1%	93.9%
		PVV:	51.9%	75.5%
		NPV:	93.4%	92.2%

Cigarette smoking: Analysis including all self-reported cigarette smokers and all non-tobacco users.
Snus use: Analysis including all self-reported snus users and all non-tobacco users.
E-cigarette use: Analysis including all self-reported E-cigarette users and all non-tobacco users.
Daily: Results from analyses excluding occasional tobacco users.
Occasional: Results from analyses excluding daily tobacco users.
Any: Results from analyses combining daily and occasional tobacco users.
κ: Cohen's kappa coefficient.
PPV: Positive predictive value
NPV: Negative predictive value

Table S4. Self-reported tobacco use against urinary cotinine levels, excluding participants with mixed tobacco use or with missing data on waterpipe use (n=117 excluded).

Self-reports	Urinary cotinine level						
**Cigarette smoking**					**Daily**	**Occasional**	**Any**
	<50 ng/ml	≥50 ng/ml	*Total*				
No	455	35	*490*	κ:	0.784	0.469	0.725
Occasional	28	36	*64*	Sensitivity:	74.8%	50.7%	80.0%
Daily	8	104	*112*	Specificity:	98.3%	94.2%	92.7%
Total	*491*	*175*	*666*	PPV:	92.9%	56.3%	79.5%
				NPV:	92.9%	92.9%	92.9%
**Snus use**					**Daily**	**Occasional**	**Any**
	<50 ng/ml	≥50 ng/ml	*Total*				
No	455	35	*490*	κ:	0.825	0.478	0.775
Occasional	22	33	*55*	Sensitivity:	77.0%	48.5%	81.1%
Daily	2	117	*119*	Specificity:	99.6%	95.4%	95.0%
Total	*479*	*185*	*664*	PPV:	98.3%	60.0%	86.2%
				NPV:	92.9%	92.9%	92.9%
**E-cigarette use**					**Any**		
	<50 ng/ml	≥50 ng/ml	*Total*				
No	455	35	*490*	κ:	0.293		
Occasional	18	7	*25*	Sensitivity:	28.6%		
Daily	0	7	*7*	Specificity:	96.2%		
Total	*473*	*49*	*522*	PPV:	43.8%		
				NPV:	92.9%		

Cigarette smoking: Analysis including self-reported cigarette smokers without other tobacco use and all non-tobacco users.
Snus use: Analysis including self-reported snus users without other tobacco use and all non-tobacco users.
E-cigarette use: Analysis including self-reported e-cigarette users without other tobacco use and all non-tobacco users.
Daily: Results from analyses excluding occasional tobacco users.
Occasional: Results from analyses excluding daily tobacco users.
Any: Results from analyses combining daily and occasional tobacco users.
κ: Cohen's kappa coefficient.
PPV: Positive predictive value
NPV: Negative predictive value

Table S5. Self-reported tobacco use against urinary cotinine levels for participants who provided urine samples within 6 weeks after answering the questionnaire (n=507 included).

Self-reports	Urinary cotinine level						
**Cigarette smoking**					**Daily**	**Occasional**	**Any**
	<50 ng/ml	≥50 ng/ml	*Total*				
No	216	15	*231*	κ:	0.867	0.639	0.785
Occasional	22	47	*69*	Sensitivity:	84.4%	75.8%	89.5%
Daily	2	81	*83*	Specificity:	99.1%	90.8%	90.0%
Total	*240*	*143*	*383*	PPV:	97.6%	68.1%	84.2%
				NPV:	93.5%	93.5%	93.5%
**Snus use**					**Daily**	**Occasional**	**Any**
	<50 ng/ml	≥50 ng/ml	*Total*				
No	216	15	*231*	κ:	0.890	0.643	0.861
Occasional	8	26	*34*	Sensitivity:	87.0%	63.4%	89.4%
Daily	1	101	*102*	Specificity:	99.5%	96.4%	96.0%
Total	*225*	*142*	*367*	PPV:	99.0%	76.5%	93.4%
				NPV:	93.5%	93.5%	93.5%
**E-cigarette use**					**Any**		
	<50 ng/ml	≥50 ng/ml	*Total*				
No	216	15	*231*	κ:	0.602		
Occasional	15	25	*40*	Sensitivity:	66.7%		
Daily	0	5	*5*	Specificity:	93.5%		
Total	*231*	*45*	*276*	PPV:	66.7%		
				NPV:	93.5%		

Cigarette smoking: Analysis including all self-reported cigarette smokers and all non-tobacco users.
Snus use: Analysis including all self-reported snus users and all non-tobacco users.
E-cigarette use: Analysis including all self-reported E-cigarette users and all non-tobacco users.
Daily: Results from analyses excluding occasional tobacco users.
Occasional: Results from analyses excluding daily tobacco users.
Any: Results from analyses combining daily and occasional tobacco users.
κ: Cohen's kappa coefficient.
PPV: Positive predictive value
NPV: Negative predictive value
