# Supplementary material for: Assessing tobacco use in Swedish young adults from self-report and urinary cotinine: a validation study using the BAMSE birth cohort
Source: BMJ Open. 2023 Jul 12;13(7):e072582. doi: 10.1136/bmjopen-2023-072582 (PMC10347476; doi:10.1136/bmjopen-2023-072582)
Supplement: Author's manuscript [file bmjopen-2023-072582.draft_revisions.pdf]

BMJ Open

BMJ Open is committed to open peer review. As part of this commitment we make the peer review history of every article we publish publicly available.

When an article is published we post the peer reviewers' comments and the authors' responses online. We also post the versions of the paper that were used during peer review. These are the versions that the peer review comments apply to.

The versions of the paper that follow are the versions that were submitted during the peer review process. They are not the versions of record or the final published versions. They should not be cited or distributed as the published version of this manuscript.

BMJ Open is an open access journal and the full, final, typeset and author-corrected version of record of the manuscript is available on our site with no access controls, subscription charges or pay-per-view fees (http://bmjopen.bmj.com).

If you have any questions on BMJ Open's open peer review process please email info.bmjopen@bmj.com

BMJ Open

Validation of self-reported tobacco use among Swedish young adults

Journal:	*BMJ Open*
Manuscript ID	bmjopen-2023-072582
Article Type:	Original research
Date Submitted by the Author:	09-Feb-2023
Complete List of Authors:	Zettergren, Anna; Karolinska Institutet, Institute of Environmental Medicine<br>Sompa, Shanzina; Karolinska Institutet, Institute of Environmental Medicine<br>Palmberg, Lena; Karolinska Institutet, Institute of Environmental Medicine<br>Ljungman, Petter; Karolinska Institutet, Institute of Environmental Medicine; Danderyd University Hospital, Department of Cardiology<br>Pershagen, Göran; Karolinska Institutet, Institute of Environmental Medicine<br>Andersson, Niklas; Institute of Environmental Medicine, Karolinska Institutet<br>Lindh, Christian ; Lund University, Division of Occupational and Environmental Medicine<br>Georgelis, Antonios; Karolinska Institutet, Institute of Environmental Medicine; Region Stockholm, Centre for Occupational and Environmental Medicine<br>Kull, Inger; Södersjukhuset AB, Sachs' Children and Youth Hospital; Karolinska Institutet, Department of Clinical Science and Education, Södersjukhuset<br>Melen, Erik; Södersjukhuset AB, Sachs' Children and Youth Hospital; Karolinska Institutet, Department of Clinical Science and Education, Södersjukhuset<br>Ekström, Sandra; Karolinska Institute; Region Stockholm, Centre for Occupational and Environmental Medicine<br>Bergstrom, Anna; Karolinska Institutet, Institute of Environmental Medicine
Keywords:	EPIDEMIOLOGY, PUBLIC HEALTH, STATISTICS & RESEARCH METHODS

For peer review only - http://bmjopen.bmj.com/site/about/guidelines.xhtml

I, the Submitting Author has the right to grant and does grant on behalf of all authors of the Work (as defined in the below author licence), an exclusive licence and/or a non-exclusive licence for contributions from authors who are: i) UK Crown employees; ii) where BMJ has agreed a CC-BY licence shall apply, and/or iii) in accordance with the terms applicable for US Federal Government officers or employees acting as part of their official duties; on a worldwide, perpetual, irrevocable, royalty-free basis to BMJ Publishing Group Ltd ("BMJ") its licensees and where the relevant Journal is co-owned by BMJ to the co-owners of the Journal, to publish the Work in this journal and any other BMJ products and to exploit all rights, as set out in our licence.

The Submitting Author accepts and understands that any supply made under these terms is made by BMJ to the Submitting Author unless you are acting as an employee on behalf of your employer or a postgraduate student of an affiliated institution which is paying any applicable article publishing charge ("APC") for Open Access articles. Where the Submitting Author wishes to make the Work available on an Open Access basis (and intends to pay the relevant APC), the terms of reuse of such Open Access shall be governed by a Creative Commons licence – details of these licences and which Creative Commons licence will apply to this Work are set out in our licence referred to above.

Other than as permitted in any relevant BMJ Author's Self Archiving Policies, I confirm this Work has not been accepted for publication elsewhere, is not being considered for publication elsewhere and does not duplicate material already published. I confirm all authors consent to publication of this Work and authorise the granting of this licence.

For peer review only

Validation of self-reported tobacco use among Swedish young adults

Anna Zettergren[1,*], Shanzina Iasmin Sompa[1], Lena Palmberg[1], Petter Ljungman[1,2], Göran Pershagen[1], Niklas Andersson[1], Christian H Lindh[3], Antonios Georgelis[1,4], Inger Kull[5,6], Erik Melén[1,5,6], Sandra Ekström[1,4], Anna Bergström[1,4]

1. Institute of Environmental Medicine, Karolinska Institutet, Stockholm Sweden

2. Department of Cardiology, Danderyd Hospital, Stockholm Sweden.

3. Division of Occupational and Environmental Medicine, Lund University, Lund, Sweden

4. Centre for Occupational and Environmental Medicine, Region Stockholm, Stockholm, Sweden

5. Sachs' Children and Youth Hospital, Södersjukhuset, Stockholm, Sweden

6. Department of Clinical Science and Education, Södersjukhuset, Karolinska Institutet, Stockholm, Sweden

*** Corresponding author:**

Anna Zettergren

Institute of Environmental Medicine, Karolinska Institutet, Stockholm Sweden.

E-mail: anna.zettergren@ki.se

Telephone: +46702022024

Word count: 4004

For peer review only - http://bmjopen.bmj.com/site/about/guidelines.xhtml

ABSTRACT

Objectives: Studies on health effects of tobacco often rely on self-reported exposure data, which is subjective and can lead to misclassification. The aim of this study was to describe the prevalence of cigarette smoking, snus and e-cigarette use, as well as to validate self-reported tobacco use among young adults in Sweden.

Method: Participants of a population-based Swedish cohort (n=3 052), aged 22-25 years, assessed their tobacco use in a web-questionnaire. Urinary cotinine was analyzed in a subsample of the study population (n=998). The agreement between self-reported tobacco use and urinary cotinine was assessed using Cohen's Kappa coefficient (κ) at a cut-off level of 50 ng/ml.

Results: Patterns of tobacco use differed between men and women. Among men, 20.0% reported daily snus use, 5.8% daily cigarette smoking and 5.6% any e-cigarette use. In contrast, 3.2% of the women reported daily snus use, 9.0% daily cigarette smoking and 2.4% any e-cigarette use. Among the tobacco use categories, daily snus users had the highest levels of cotinine. Of reported non-tobacco users, 3.5% had cotinine levels above the cut-off, compared to 68.0% among both occasional cigarette smokers and snus users, 67.5% among all e-cigarette users and 94.7% and 97.8% among daily cigarette smokers and snus users, respectively. Agreement between self-reported tobacco use and urinary cotinine was classified as strong for daily use of cigarettes (κ =0.824) and snus (κ=0.861), while moderate to weak for occasional smoking (κ=0.618), occasional snus use (κ=0.573) and any e-cigarette use (κ=0.576).

Conclusions: We found high validity of self-reported tobacco use in our study population, particularly for daily tobacco use. Further we found that daily snus users were exposed to high levels of cotinine. Together with previous findings, our results indicate good validity of self-reported tobacco use among young adults.

For peer review only - http://bmjopen.bmj.com/site/about/guidelines.xhtml

STRENGTH AND LIMITATIONS OF THIS STUDY

- Several types of tobacco use were validated using biochemical verification.
- This is the first study to validate snus use in a population of young adults comprised of both men and women.
- A large study sample including both young men and women was analyzed.
- The study was conducted within a well characterized, population-based birth cohort.
- A time lag existed between reporting tobacco use and biochemical verification.

For peer review only

For peer review only - http://bmjopen.bmj.com/site/about/guidelines.xhtml

INTRODUCTION

Research on health effects of tobacco mainly relies on self-reported exposure data. This type of exposure assessment is subjective and may result in under- or overreporting(1). Objective assessment of tobacco use can be performed using cotinine, a metabolite of nicotine, as a biomarker in urine, blood or saliva(2). However, these methods are expensive and time consuming, and are therefore not feasible for large populations. Instead, biomarker data can be used to validate self-reported data in smaller subpopulations, to estimate validity in larger cohorts.

Self-reported cigarette smoking has been validated in several studies with varying results in different populations, but with generally high validity(1). However, for other types of tobacco and nicotine products, such as smokeless tobacco and electronic cigarettes (e-cigarettes), validation studies are scarce(3). In Sweden, the smokeless tobacco type snus is used to a higher extent than cigarettes(4). While cigarette smoking has decreased in the Swedish adult population over the last several decades, to 6% daily smokers in 2022, daily snus consumption rates have increased to a current 14%, with the largest increases being among younger populations(4). Men have a notably higher consumption of snus compared to women in Sweden, however women's consumption is increasing rapidly. Although snus share some active compounds with cigarettes, much less is known about its health effects, particularly among young adults(5). Self-reported snus use has been validated among Swedish pregnant women, where high agreement between self-reported snus use and urinary cotinine levels was found(6). Still, no study has investigated the validity of self-reported information on snus use among young men and non-pregnant women.

E-cigarette use has increased globally the past decade, especially among young populations. In Sweden, an estimated 2% of the population use e-cigarettes, with the highest prevalence among men under 30 years(4,7). Although initially intended to reduce the harmful effects of cigarettes by facilitating smoking cessation, e-cigarettes are currently under debate as to whether they rather cause adverse health effects among adolescents and young adults(8). Still, more research is needed to evaluate potential health effects of e-cigarettes(9).

For peer review only - http://bmjopen.bmj.com/site/about/guidelines.xhtml

With the need for research on health effects of tobacco and nicotine products among young adults comes the need for reliable exposure data. Further, as validity of self-reported tobacco use may change over time and be influenced by factors such as age and gender, as well as smoking habits and attitudes towards tobacco products, it is important to regularly perform validation studies in different populations. Therefore, the aims of this study were to describe the prevalence of cigarette smoking, snus use, and e-cigarette use as well as to validate self-reported use of these tobacco types using urinary cotinine among young adults in a Swedish cohort.

METHODS

Study population

This cross-sectional study was conducted within the population-based Swedish birth cohort BAMSE. Detailed information on the BAMSE study is provided elsewhere(10,11). The cohort consists of 4089 newborn children recruited between 1994 and 1996 in Stockholm, Sweden. The participants have been followed up with repeated questionnaires on lifestyle and health up to approximately 24 years of age. On several occasions, participants who answered the questionnaire were invited to clinical examinations. At the follow up at around age 24, hereafter called the 24-year follow-up, conducted between December 2016 and May 2019, the response rate of the questionnaire and the attendance rate at the clinical examination were 75% (n=3 064) and 56% (n=2 270) of the original cohort, respectively.

The study was approved by the Regional Ethics Committee at Karolinska Institutet in Stockholm, Sweden and conducted according to the Helsinki Declaration. All participants provided written informed consent.

Patient and public involvement

Participants or the public were not involved in the design, conduct, reporting, or dissemination plans or the study.

Self-reported tobacco use

For peer review only - http://bmjopen.bmj.com/site/about/guidelines.xhtml

During the 24-year follow-up, participants provided self-reported information on tobacco consumption in a web-questionnaire. The current study included all participants who provided information on use of cigarettes, snus, and e-cigarettes (n=3 052), hereafter referred to as the study population. Participants were asked if they smoked cigarettes (answer options: "No"; "No, but I used to smoke"; "Yes, sometimes"; "Yes, daily"), used snus ("No"; "No, but I used to use snus"; "Yes, sometimes"; "Yes, daily"), smoked e-cigarettes ("No"; "Yes, sometimes"; "Yes, daily") or waterpipe ("No"; "Yes, every month"; "Yes, every week"; "Yes, daily"). For cigarettes and snus, the participants were asked to estimate average consumption per day (for daily smokers), week (for daily snus users and occasional cigarette smokers) or month (for occasional snus users) using open ended questions. Age of regular use debut (at least once per week) of snus or cigarettes was also assessed. In addition, participants were asked whether they had used nicotine replacement products on the day of urine sampling.

Validation population

To validate self-reported tobacco use, a subsample of the BAMSE 24-year follow-up was chosen for urine analysis of cotinine. The selection was conducted among participants who attended the clinical examination, and included all who reported daily smoking, daily or occasional snus use, daily or occasional e-cigarette use as well as all participants who provided a urine sample at the 4-year follow-up. The selection criteria resulted in 429 men and 439 women. An additional random selection of participants who provided a urine sample at 24-years was conducted to reach a subsample of 1000 participants, (500 men and 500 women). One participant with missing information on cigarette use and one participant who reported use of nicotine replacement products on the day of urine sampling were excluded, resulting in a validation population size of n=998. See **Figure S1** in the supplement for an overview of the selection process.

Cotinine analysis

Urine samples collected at the clinical examination during the 24-year follow up were analyzed for cotinine at the Division for Occupational and Environmental Medicine at Lund University. Samples were stored in -80°C until analysis. Urinary cotinine was analyzed using a modified method for

For peer review only - http://bmjopen.bmj.com/site/about/guidelines.xhtml

serum(12) using liquid chromatography tandem mass spectrometry (LC-MS/MS; QTRAP 5500, Framingham, MA, USA). Briefly, urine samples were prepared in 96-well plates, diluted in buffer and hydrolyzed using β-glucuronidase after deuterium-labelled internal standard was added. Samples were analyzed randomized, and each batch was analyzed with calibration standards, quality controls and chemical blank samples (Milli-Q water). The limit of detection (LOD) for cotinine was set to 1 ng/ml. Specific gravity (SG) was used to compensate for urine sample dilution and was measured using a digital refractometer. The analysis of cotinine is part of a quality control program between analytical laboratories coordinated by the University of Erlangen-Nuremberg, Germany.

Statistical methods

Comparisons of background characteristics and tobacco use between men and women were performed with two-tailed t-tests, Wilcoxon rank-sum test or Pearson χ^2 test, as appropriate. The average consumption of cigarettes was categorized as <10, ≥10 cigarettes per day for daily smokers, based on an *a priori* decided cut-off. Snus consumption was categorized as <4 and ≥4 boxes of snus per week for daily snus users, based on sample distribution. Cotinine levels were adjusted for SG according to: $PSG=P * [(1.014) / (SG-1)]$, where *PSG* is the adjusted cotinine concentration, *P* is the detected cotinine concentration (ng/ml), 1.014 is the average SG in the validation population and *SG* is the specific gravity of the individual urine sample(13). Cotinine levels between tobacco types and gender were compared using Wilcoxon rank-sum test.

To assess the agreement between self-reported tobacco use and urinary cotinine levels, Cohen's Kappa coefficient was calculated at a cut-off level of 50 ng/ml cotinine, as recommended by a subcommittee of the Society for tobacco and nicotine Research(14). Kappa coefficients were interpreted according to McHugh (2012) (15) as 0.00–0.20: no agreement, 0.21–0.39: minimal agreement, 0.40–0.59: weak agreement, 0.60–0.79: moderate agreement, 0.80–0.90: strong agreement, and >0.90: almost perfect agreement. Sensitivity, specificity, positive predictive value (PPV) and negative predictive value (NPV) were also investigated. Analyses were stratified by type of tobacco (cigarettes, snus or e-cigarettes), daily or occasional use and gender. The use of waterpipe was not validated due to few users, but users of waterpipe were considered as tobacco users. Sensitivity analyses were performed

For peer review only - http://bmjopen.bmj.com/site/about/guidelines.xhtml

For peer review only

excluding participants with mixed tobacco use (i.e. using more than one tobacco type) or with missing data on waterpipe use (n=117). Another set of analysis was performed excluding those who provided the urine sample more than 6 weeks after answering the web-questionnaire (n=492).

A prediction model was performed using logistic regression to assess factors associated with false negative reports of tobacco use, so-called underreporting (i.e., reporting no tobacco use but having a urine sample with ≥50 ng/ml cotinine). Factors included in the final model were identified using a stepwise backward selection approach according to Kirkwood & Sterne(16), eliminating factors at a threshold level of $p<0.2$ from a log-likelihood ratio test. Factors considered were identified from the literature and included gender, parental socioeconomic status and parental smoking obtained from the baseline questionnaire; educational level, occupation, asthma, former smoking, former snus use, secondhand smoke exposure, body mass index (BMI), weekday of urine sampling and time between web-questionnaire and urine sampling obtained from the 24-year follow-up. For details on covariate definitions, see supplement. Participants with missing data were excluded from the analyses. Statistical analyses were performed in STATA (version 16; Stata Corp, College Station, TX, USA). Statistical significance was considered at $p<0.05$.

RESULTS

Description of study population

An overview of background characteristics of the study population can be seen in **Table 1**. The mean age was 22.5 years (range 21.5–25.2 years), 53.0% were women and most participants were students (51.2%), followed by employed workers (40.9%) and other occupations (7.9%). The median time between answering the questionnaire and the urine sampling was 41 days (interquartile range 16–84 days).

For peer review only - http://bmjopen.bmj.com/site/about/guidelines.xhtml

Table 1. Characteristics of the study population and the validation population at the BAMSE 24-year follow-up.

	Study population n=3052	Validation population n=998
	**n (%)**	**n (%)**
Male	1 435 (47.0)	499 (50.0)
White collar at baseline	2 537 (84.5)	811 (82.6)
Any parent smoking at baseline	618 (20.4)	209 (21.1)
Asthma	345 (11.3)	137 (13.7)
University degree at age 24	1 093 (36.0)	360 (36.2)
Main occupation:		
Student	1 561 (51.2)	486 (48.7)
Working	1 246 (40.9)	438 (43.9)
Other	240 (7.9)	74 (7.4)
	**Mean (SD)**	**Mean (SD)**
Age, years	22.5 (0.6)	22.4 (0.4)
BMI[a], kg/m^2	23.1 (3.9)	23.4 (4.1)
	**Median (IQR)**	**Median (IQR)**
Time between questionnaire and urine sampling[a]	41 (16-84)	42 (16-78)

Percentages may not add up due to missing values.
[a]: Data available from participants who attended the clinical examination at the 24-year follow-up, 74% (n=2264) of study population
SD: Standard deviation
IQR: Interquartile range

Self-reported tobacco use

Self-reported tobacco use is presented in **Table 2**. Smoking was reported by 20.7% (7.5% daily and 13.3% occasionally), snus use by 15.6% (11.1% daily and 4.5% occasionally) and e-cigarettes by 3.9% (0.5% daily and 3.4% occasionally). Women reported daily cigarette smoking to a larger extent than men, while men reported higher snus and e-cigarette use. A total of 34.3% of the participants reported some type of tobacco use, and among these, 20.8% used more than one type. Among daily smokers, the average number of cigarettes per day was 8.6 among women and 9.7 among men (p=0.085). The average number of boxes per week among daily snus users was significantly higher among men than women (3.0 vs. 1.8, p=0.003). Women began to smoke cigarettes regularly at a younger age than men, while men were younger when beginning to use snus regularly. Secondhand smoke exposure was reported by 3.1% (5.7% among tobacco users and 1.9% among non-users).

For peer review only - http://bmjopen.bmj.com/site/about/guidelines.xhtml

For peer review only

Table 2. Self-reported tobacco use in the BAMSE cohort, at the 24-year follow up.

	All n=3 052	Women n=1 617	Men n=1 435	p-value[a] (women vs. men)
	n (%)			
**Cigarettes**				
Never	2 037 (66.7)	1 056 (65.3)	981 (68.4)	0.074
Former	382 (12.5)	196 (12.1)	186 (13.0)	0.484
Occasional	405 (13.3)	220 (13.6)	185 (12.9)	0.562
Daily	228 (7.5)	145 (9.0)	83 (5.8)	0.001
<10 cigarettes/day	*122 (54.2)*	*84 (58.3)*	*38 (46.9)*	*0.099*
≥10 cigarettes/day	*103 (45.2)*	*60 (41.4)*	*43 (51.8)*	
**Snus**				
Never	2 459 (80.6)	1 481 (91.6)	978 (68.2)	<0.0001
Former	116 (3.8)	27 (1.7)	89 (6.2)	<0.0001
Occasional	138 (4.5)	57 (3.5)	81 (5.6)	0.005
Daily	339 (11.1)	52 (3.2)	287 (20.0)	<0.0001
<4 boxes of snus/week	*184 (54.4)*	*34 (65.4)*	*150 (52.4)*	*0.085*
≥4 boxes of snus/week	*154 (45.6)*	*18 (34.6)*	*136 (47.6)*	
**E-cigarettes**				
No	2 932 (96.1)	1 578 (97.6)	1 354 (94.4)	<0.0001
Occasional	105 (3.4)	35 (2.2)	70 (4.9)	<0.0001
Daily	15 (0.5)	4 (0.3)	11 (0.8)	0.041
**Waterpipe**				
No	2 835 (97.9)	1 533 (97.9)	1 302 (97.8)	0.997
Occasional	60 (2.1)	32 (2.0)	28 (2.1)	0.895
Daily	2 (0.1)	1 (0.1)	1 (0.1)	0.910
**Number of tobacco types used**				
1	830 (79.2)	406 (85.7)	424 (73.9)	<0.0001
>1	218 (20.8)	69 (14.3)	150 (26.1)	
**Secondhand smoke exposure**				
Yes[1]	89 (3.1)	49 (3.2)	40 (3.1)	0.852
	Mean (range)			p-value
Age when starting to smoke cigarettes regularly	17.1 (12-22)	16.9 (12-22)	17.4 (12-22)	0.013[b]
Mean nr cigarettes/week (occasional smokers)	8.3 (1-150)	7.7 (0-50)	9.0 (0-150)	0.856[c]
Mean nr cigarettes/day (daily smokers)	9.0 (1-30)	8.6 (1-25)	9.7 (1-30)	0.085[c]
Age when starting to snus regularly	18.5 (13-24)	20.2 (15-23)	18.0 (13-24)	<0.0001[b]
Mean nr boxes snus/month (occasional snus users)	2.5 (0-15)	1.8 (0-10)	3.0 (0-15)	0.003[c]
Mean nr boxes snus/week (daily snus users)	3.6 (0.5-10)	3.0 (0.5-7)	3.7 (0.5-10)	0.004[c]

[a]: P-value from Pearson χ^2 test.
[b]: P-value from two-tailed t-test.
[c]: P-value from Wilcoxon rank-sum test
[1]: Defined as daily exposure to indoor tobacco smoke

For peer review only - http://bmjopen.bmj.com/site/about/guidelines.xhtml

Description of validation population

The background characteristics of the validation population did not differ from the total study population (**Table 1**), apart from a slightly higher prevalence of men (50.0% vs. 47.0%) and current asthma (13.7% vs 11.3%). As a consequence of the selection criteria, self-reported tobacco use in the validation population was higher than in the total study population, with 51.0% using any tobacco (daily or occasionally). A summary of tobacco use in the validation population is displayed in **Table S1** in the supplement.

Urine cotinine levels

Cotinine levels by tobacco type is shown in **Figure 1** and detailed urine cotinine levels are presented in **Table S2** in the supplement. In total, 76.2% of the samples had cotinine levels above the LOD and 44.4% had levels over the 50 ng/ml cut-off for validation. For all types of tobacco, daily users had significantly higher median cotinine levels than occasional users; 2 094 ng/ml vs. 422 ng/ml ($p<0.0001$) for smokers, 3 599 ng/ml vs. 345 ng/ml ($p<0.0001$) for snus users and 2 749 ng/ml vs. 527 ng/ml ($p=0.019$) for e-cigarette users. This pattern remained after exclusion of participants with mixed tobacco use, although cotinine levels were generally lower in each group. Among mixed tobacco users the median cotinine level was 3 073 ng/ml. Significantly higher cotinine levels were observed among high compared to low consumption of daily cigarette smoking and snus use (≥10 cigarettes/day or ≥ 4 boxes/week) after excluding other tobacco use (**Figures S2 and S3**). Men had higher cotinine levels than women among occasional cigarette smokers (1 595 ng/ml vs. 159 ng/ml, $p=0.0003$) and daily snus users (3 900 ng/ml vs. 2 762 ng/ml, $p=0.006$). However, no significant gender differences were observed after excluding mixed tobacco users. The median level for non-users was 1.6 ng/ml. Non-tobacco users exposed to daily secondhand smoke (n=7) had a median cotinine level of 2.47 ng/ml.

Validation of self-reported tobacco use

Table 3 displays validation statistics of self-reported tobacco use against urinary cotinine levels, by category of tobacco use. The overall agreement was 86.5%, the overall false negative rate (i.e., underreporting) was 3.5% and the overall false positive rate (i.e., overreporting) was 10.0%. Among

For peer review only - http://bmjopen.bmj.com/site/about/guidelines.xhtml

the underreporters, 40.0% reported former tobacco use and among the overreporters, 88.0% reported occasional tobacco use.

For both daily cigarette smoking and snus use, the agreement between self-reports and urinary cotinine was classified according to McHugh as strong (κ=0.824 and κ=0.861, respectively), with high sensitivity (80.2% and 83.6%, respectively) and specificity (98.3% and 99.1%, respectively). For occasional smoking and snus use, agreement and sensitivity were lower at the chosen cut-off level (κ=0.618 and κ=0.573, respectively), while specificity remained high. For any use of cigarettes and snus (daily and occasional combined) the agreement, sensitivity and specificity were high, with moderate agreement for smoking (κ=0.762) and strong for snus (κ=0.814). For any e-cigarette use, the agreement was weak (κ=0.576), with lower sensitivity (60.7%) compared to any smoking (86.6%) and any snus use (86.8%), but with high specificity (94.6%). No separate analyses were made for daily or occasional use for e-cigarettes, due to few daily users. However, all daily users of e-cigarettes had cotinine levels above the 50 ng/ml cut-off. NPV was consistently high across tobacco categories, while PPV was high for daily tobacco use (94.7% for cigarettes and 97.8% for snus) and somewhat lower for occasional use (68.0% for cigarettes and snus) and any e-cigarette use (67.5%).

Stratified analyses by gender revealed higher validity among men for snus and e-cigarette use, while no major gender differences were observed for cigarette smoking (see supplement **Table S3** for details).

Sensitivity analyses

When excluding mixed tobacco users, agreement was generally somewhat lower compared to the analyses with the full validation population (see supplement **Table S4** for details). In contrast, the agreement tended to be somewhat higher after exclusion of participants who provided urine samples ≥6 weeks after answering the questionnaire, however the interpretation of Kappa values was generally not influenced (see **Table S5** for details).

For peer review only

For peer review only - http://bmjopen.bmj.com/site/about/guidelines.xhtml

Table 3. Self-reported tobacco use against urinary cotinine levels by tobacco use.

Self-reports	Urinary cotinine level						
**Cigarette smoking**					**Daily**	**Occasional**	**Any**
	<50 ng/ml	≥50 ng/ml	*Total*	κ:	0.824	0.618	0.762
No	455	35	*490*	Sensitivity:	80.2%	70.8%	86.6%
Occasional	40	85	*125*	Specificity:	98.3%	91.9%	90.5%
Daily	8	142	*150*	PPV:	94.7%	68.0%	82.5%
Total	*503*	*262*	*765*	NPV:	92.9%	92.9%	92.9%
**Snus use**					**Daily**	**Occasional**	**Any**
	<50 ng/ml	≥50 ng/ml	*Total*	κ:	0.861	0.573	0.814
No	455	35	*490*	Sensitivity:	83.6%	59.3%	86.8%
Occasional	24	51	*75*	Specificity:	99.1%	95.0%	94.2%
Daily	4	178	*182*	PPV:	97.8%	68.0%	89.1%
Total	*483*	*264*	*747*	NPV:	92.9%	92.9%	92.9%
**E-cigarette use**					**Any**		
	<50 ng/ml	≥50 ng/ml	*Total*	κ:	0.576		
No	455	35	*490*	Sensitivity:	60.7%		
Occasional	26	45	*71*	Specificity:	94.6%		
Daily	0	9	*9*	PPV:	67.5%		
Total	*481*	*89*	*570*	NPV:	92.9%		

Cigarette smoking: Analysis including all self-reported cigarette smokers and all non-tobacco users.
Snus use: Analysis including all self-reported snus users and all non-tobacco users.
E-cigarette use: Analysis including all self-reported e-cigarette users and all non-tobacco users.
Daily: Results from analyses excluding occasional tobacco users.
Occasional: Results from analyses excluding daily tobacco users.
Any: Results from analyses combining daily and occasional tobacco users.
κ: Cohen's kappa coefficient.
PPV: Positive predictive value
NPV: Negative predictive value

Prediction model for underreporting

Using the backward stepwise selection approach, the factors former smoking, former snus use and time between questionnaire and urine sampling were included in the final prediction model. Former snus users were more likely to underreport tobacco use (odds ratio (OR): 5.7, 95% CI: 2.2, 15,0), and a non-significant association was observed for former smoking (OR: 2.1, 95% CI: 0.9, 4.6). Providing the urine sample more than 6 weeks after the questionnaire, as compared to within 6 weeks after the

For peer review only

For peer review only - http://bmjopen.bmj.com/site/about/guidelines.xhtml

questionnaire, was also predictive of underreporting (OR: 1.5, 95% CI: 0.7, 2.9), although not statistically significant.

DISCUSSION

Among young adults from a Swedish population-based cohort study, we observed high validity of self-reported tobacco use, particularly among daily users, while somewhat lower for occasional use as well as for e-cigarette use at the cut-off level of 50 ng/ml urinary cotinine. Specificity and NPV of the questionnaire were consistently high across tobacco type and frequency of use, while sensitivity and PPV varied and was somewhat lower, especially for occasional tobacco use. Former use of tobacco and a longer time between answering the questionnaire and providing a urine sample were predictive of underreporting, although former snus use was the only statistically significant prediction factor. Further, there were significant gender differences in tobacco use. Daily consumption of snus was high among men, while daily cigarette smoking was more common among women. Daily e-cigarette use was uncommon among both sexes. Daily snus users had the highest urine cotinine levels.

This study has several strengths, including a large and well-characterized study population from a population-based cohort, which provided power for the statistical analyses and allowed for stratification by tobacco type and gender. Furthermore, the study had repeatedly collected information on tobacco exposure, as well as sociodemographic factors. However, a limitation of the study design is that the questionnaire and the urine sampling were not conducted at the same occasion, and some participants may have changed their tobacco habits between the two assessments. The importance of time between the two assessments was apparent from both the sensitivity analysis as well as from the prediction model. Another limitation is the lack of information specific tobacco brands or types, such as nicotine-free products, which may further cause misclassification of certain self-reported tobacco users. Similarly, use of nicotine replacement products may explain cotinine levels above the cut-off level among some of the former tobacco users(17). However, only one participant in the validation populations reported using nicotine replacement products at the day of urine sampling and was thus excluded from the analyses.

For peer review only

Our study shows similar prevalence of tobacco use among young adults compared to data for the age group 16-29 years from Swedish national surveys conducted in 2016, 2018 and 2020(18,19). From 2016-2020, daily smoking was reported by 4-8% of young adults, daily snus use by 10-11% and any e-cigarette use at 2-3%, as compared to 7%, 11%, and 4%, respectively, in our study. On the other hand, occasional smoking was somewhat higher in our study (13% vs. 8-11%). Further, the observed gender differences in our study were similar to those of a recent study among Swedish 19-yearolds(20) and with figures from the Swedish national survey, describing higher snus and e-cigarette use among young men compared to women, but no difference in smoking prevalence between the genders(18,19). However, a recent study on pooled data from two population based Swedish cohort also found that cigarette smoking was more common among women across the age group 20-75 years(7).

Urinary cotinine levels were consistent with self-reported frequency of tobacco use. Daily users had higher cotinine levels than occasional users, and a dose-response trend was observed for the amount of cigarettes and snus consumed. Daily snus users had the highest median cotinine levels, followed by e-cigarette and cigarette users. Smokeless tobacco users have previously been found to have higher urinary cotinine levels than cigarettes smokers(21,22), while the difference between cigarette smokers and e-cigarette users appear less consistent(22–24). However, e-cigarette users were few in our study and the resulting cotinine levels may not be generalizable to other populations.

The high validity of self-reported tobacco use found in our study is consistent with previous findings. Several validation studies on self-reported cigarette smoking have found high correlation between urinary cotinine and self-reported smoking status in population-based cohorts, including young adult populations(25–30). In a cohort of young men and women in the United States (aged 18-30 years), the overall agreement between self-reported cigarette smoking (at least 5 cigarettes per week) and urinary cotinine at a cut-off level of 14 ng/ml was 95.8%(30). Similarly, high sensitivity (93%) and specificity (99%) for self-reported smoking compared to a cotinine cut-off level of 25 ng/ml was found in a cohort of Malaysian male university students(29).

In a previous validation study of self-reported snus use, high validity was found among Swedish pregnant women and adolescents (mean age 15 years)(6,31).

For peer review only - http://bmjopen.bmj.com/site/about/guidelines.xhtml

To date, few studies have validated self-reported e-cigarette use against biomarkers in population-based cohorts. Boykan et al.(32) found a low (37%) concordance among adolescent self-reported e-cigarette users in the past week. The lower agreement observed for self-reported e-cigarette use in our study as compared to cigarettes and snus is likely a consequence of that most of the e-cigarette use was occasional, which was also true in the study by Boykan et al. In fact, in our study all daily e-cigarette users had cotinine levels above the cut-off value, although they were very few. Further studies with larger sample sizes are needed to assess validity of self-reported e-cigarette use more confidently. Some discrepancies between genders were observed for validity of self-reporting where men had generally higher validity, particularly for snus and e-cigarette use where the prevalence was higher among men. However, gender was not a predictive factor for underreporting. In studies conducted in the United Arab Emirates, Georgia and South Korea, much lower validity for self-reported smoking was found among women compared to men, however smoking rates were also much lower among women it these studied(25,33,34). In contrast, in studies from Canada, the United States and Mexico, where smoking rates were more even between genders, similar validity was also observed(28,30,35). These discrepancies may be related to cultural differences between the geographic regions of the studies.

We found low rates of underreporting in our study population, in a similar or lower range than other study populations from the general population(25–28,30,31,36). However, it should be noted that rates of underreporting tend to be notably higher among certain patient groups, for example chronic obstructive pulmonary disease patients(37), head and neck cancer patients(38,39), cardiovascular disease patients(40,41), as well as among pregnant women(6,42,43), especially when investigating smoking cessation. Although some nicotine exposure may occur from secondhand smoke exposure or diet(44,45), such exposure is unlikely to cause misclassification at a cut-off level 50 ng/ml. Instead, the strongest predictive factor of underreporting was former tobacco use. This is in line with previous findings(28,30), and may be explained by relapse or use of other nicotine products during the study period. Some overreporting was also found in the validation population, particularly among occasional tobacco users. Lower agreement in this group is expected compared to daily tobacco users at the same

For peer review only - http://bmjopen.bmj.com/site/about/guidelines.xhtml

biochemical cut-off level, as their nicotine exposure would vary more over time. The same discrepancies between these groups and similar level of agreement between self-reported occasional cigarette smoking and serum cotinine was observed in a recent NHANES study(36). As the half-life of cotinine is 12-20 hours(2), it is likely that occasional users will have urinary cotinine levels similar to non-users only a few days after their latest use. Validity among occasional tobacco users is therefore likely not fully captured with a single urine sampling or at the chosen cut-off level.

Despite being enriched with tobacco users, the validation population was generally representative of the study population in demographic factors, which indicates high validity in the whole cohort. However, the different proportions of tobacco user, and subsequently non-tobacco users, means that the level of over- and underreporting might differ. As there were proportionally more non-tobacco users in the study population than the validation population, the rate of underreporting may be somewhat higher. Similarly, the number of overreporters in the study population may be lower, due to tobacco users being proportionally fewer. Still, the results of the validation analyses will be applicable to future research conducted on tobacco exposure in the BAMSE cohort. From a broader perspective, these results together with previous findings indicate that self-reported data on tobacco consumption can be used to accurately assess exposure for young adults. Questionnaires are important tools to study health effects of tobacco and can be used to meet the demand on more research on both e-cigarettes and snus. Although snus is banned in most EU countries(46) and is not a globally used type of tobacco, there has been a rise in snus sales in the USA over the past years(47). This spread on the market increases the need to uncover adverse health effects of snus, in order to promote global public health.

CONCLUSION

In this study, we observed significant gender differences in tobacco use among Swedish young adults, with a notably high prevalence of daily snus consumption among young men. Snus users had the highest levels of urinary cotinine. Further, we found high validity of self-reported tobacco use, particularly among daily tobacco users, with very low rates of underreporting. These results indicate

For peer review only

For peer review only - http://bmjopen.bmj.com/site/about/guidelines.xhtml

that self-reported tobacco use is valid for exposure assessment among young adults, which is important for future research on health effects of tobacco and nicotine in this age group.

List of abbreviations

E-cigarettes: Electronic cigarettes

LC-MS/MS: Liquid chromatography tandem mass spectrometry

BMI: Body mass index

LOD: Limit of detection

SG: Specific gravity

PPV: Positive predictive value

NPV: Negative predictive value

OR: Odds ratio

For peer review only

For peer review only - http://bmjopen.bmj.com/site/about/guidelines.xhtml

Acknowledgement

We thank the children and parents participating in the BAMSE cohort and all staff involved in the study through the years. Moreover, we wish to thank Anna Rönnholm, Marie Bengtsson, and Åsa Amilon at the Division of Occupational and Environmental Medicine at Lund University, Sweden, for performing the analyses of chemicals.

Funding

This work was supported by the Swedish Research Council (VR, grant number 2018-02524); the Swedish Heart and Lung Foundation; the Swedish Research Council for Sustainable Development (FORMAS, grant number 2016-01646); the Swedish Research Council for Working Life and Social Welfare; the Swedish Asthma and Allergy Association Research Foundation; the Swedish Environmental Protection Agency (grant numbers NV-09284-13 and NV-00175-15); Region Stockholm (ALF project, and for cohort and database maintenance); the European Research Council (grant number 757919); and Karolinska Institutet.

Competing interests

The authors declare that they have no competing interests.

Author's contribution

AZ took part in the study design, performed the formal analyses and interpretation of the results, created tables and figures, and wrote the original draft. SS contributed to revising the manuscript. LP contributed to revising the manuscript and provided funding. PL contributed to revising the manuscript and provided funding. GP contributed to revising the manuscript. NA provided data curation, assistance with the formal analysis and contributed to revising the manuscript. CL provided resources for and was responsible for the urine analysis and contributed to revising the manuscript. AG contributed to revising the manuscript. IK contributed to revising the manuscript. EM contributed to revising the manuscript and provided funding. SE took part in the study design, provided data curation and assistance with the formal analysis, contributed to the supervision of the project was a major contributor in revising the manuscript. AB had a major role in study design, methodology, project

For peer review only - http://bmjopen.bmj.com/site/about/guidelines.xhtml

For peer review only

administration and supervision of the project, provided funding and resources and was a major contributor in revising the manuscript. All authors read and approved the final manuscript.

Availability of data and materials

The datasets generated and/or analyzed during the current study are not publicly available, due to the dataset containing sensitive personal data, but are available from the corresponding author on reasonable request.

Ethical approval statement

The study was approved by the Regional Ethics Committee at Karolinska Institutet in Stockholm, Sweden (Dnr 2016/1380-31/2).

References

1. Gorber SC, Schofield-Hurwitz S, Hardt J, Levasseur G, Tremblay M. The accuracy of self-reported smoking: A systematic review of the relationship between self-reported and cotinine-assessed smoking status. Nicotine Tob Res. 2009;11(1):12–24.
2. Kim S. Overview of cotinine cutoff values for smoking status classification. Int J Environ Res Public Health. 2016;13(12).
3. Agaku IT, King BA. Validation of Self-Reported Smokeless Tobacco Use by Measurement of Serum Cotinine Concentration Among US Adults. Am J Epidemiol. 2014;180(7):749–54.
4. Swedish Public Health Agency. Vuxnas bruk av tobaks- och nikotinprodukter [Internet]. 2022 [cited 2023 Jan 25]. Available from: https://www.folkhalsomyndigheten.se/livsvillkor-levnadsvanor/andts/utveckling-inom-andts-anvandning-och-ohalsa/anvandning/anvandning-av-tobaks-och-nikotinprodukter/vuxnas-bruk-av-tobaks--och-nikotinprodukter/
5. Siddiqi K, Husain S, Vidyasagaran A, Readshaw A, Mishu MP, Sheikh A. Global burden of disease due to smokeless tobacco consumption in adults: An updated analysis of data from 127

For peer review only

For peer review only - http://bmjopen.bmj.com/site/about/guidelines.xhtml

countries. BMC Med. 2020;18(1).

6. Gunnerbeck A, Raaschou P, Cnattingius S, Edstedt Bonamy AK, Wickström R. Maternal snuff use and cotinine in late pregnancy—A validation study. Acta Obstet Gynecol Scand. 2018;97(11):1373–80.

7. Hedman L, Backman H, Stridsman C, Bosson JA, Lundbäck M, Lindberg A, et al. Association of Electronic Cigarette Use With Smoking Habits, Demographic Factors, and Respiratory Symptoms. JAMA Netw open. 2018;1(3):e180789.

8. Rom O, Pecorelli A, Valacchi G, Reznick AZ. Are E-cigarettes a safe and good alternative to cigarette smoking? Ann N Y Acad Sci. 2015;1340(1):65–74.

9. Gotts JE, Jordt SE, McConnell R, Tarran R. What are the respiratory effects of e-cigarettes? BMJ. 2019;366:1–31.

10. Melén E, Bergström A, Kull I, Almqvist C, Andersson N, Asarnoj A, et al. Male sex is strongly associated with IgE-sensitization to airborne but not food allergens: Results up to age 24 years from the BAMSE birth cohort. Clin Transl Allergy [Internet]. 2020;10(1):1–7. Available from: https://doi.org/10.1186/s13601-020-00319-w

11. Wickman M, Kull I, Pershagen G, Sl N, Bamse T. The BAMSE Project : presentation of a prospective longitudinal birth cohort study. Pediatr Allergy Immunol. 2002;13:11–3.

12. Axelsson J, Rylander L, Rignell-Hydbom A, Lindh CH, Jönsson BAG, Giwercman A. Prenatal phthalate exposure and reproductive function in young men. Environ Res [Internet]. 2015;138:264–70. Available from: http://dx.doi.org/10.1016/j.envres.2015.02.024

13. Hauser R, Meeker JD, Park S, Silva MJ, Calafat AM. Temporal variability of urinary phthalate metabolite levels in men of reproductive age. Environ Health Perspect. 2004;112(17):1734–40.

14. Benowitz NL, Jacob P, Ahijevych K, Jarvis MJ, Hall S, LeHouezec J, et al. SRNT Subcommittee on Biochemical Verification. Biochemical verification of tobacco use and cessation. Nicotine Tob Res. 2002;4(2):149–59.

For peer review only - http://bmjopen.bmj.com/site/about/guidelines.xhtml

15. McHugh ML. Interrater reliability: the kappa statistics. Biochem Medica. 2012;22(3):276–82.

16. Kirkwood BR, Sterne JAC. Essential Medical Statistics. 2nd ed. Malden, Massachusetts: Blackwell Publishing Company; 2003. 341 p.

17. Hickson C, Lewis S, Campbell KA, Cooper S, Berlin I, Claire R, et al. Comparison of nicotine exposure during pregnancy when smoking and abstinent with nicotine replacement therapy: systematic review and meta-analysis. Addiction. 2019;114(3):406–24.

18. Swedish Public Health Agency. Vuxnas bruk av cigaretter, snus och e-cigaretter [Internet]. 2020 [cited 2020 Nov 13]. Available from: https://www.folkhalsomyndigheten.se/livsvillkor-levnadsvanor/andts/utveckling-inom-andts-anvandning-och-ohalsa/bruk/tobak-och-liknande-produkter/vuxnas-bruk-av-cigaretter-snus-och-e-cigaretter/

19. Swedish Public Health Agency. Tobakskonsumtion (självrapporterat) efter ålder, kön och år. Andel (procent). [Internet]. 2020 [cited 2021 May 3]. Available from: http://fohm-app.folkhalsomyndigheten.se/Folkhalsodata/pxweb/en/B_HLV/B_HLV__aLevvanor__aagLevvanortobak/hlv1tobaald.px/

20. Hedman L, Backman H, Stridsman C, Lundbäck M, Andersson M, Rönmark E. Predictors of electronic cigarette use among Swedish teenagers: A population-based cohort study. BMJ Open. 2020;10(12).

21. Çok I, Öztürk R. Urinary cotinine levels of smokeless tobacco (Maraş powder) users1. Hum Exp Toxicol. 2000;19(11):650–5.

22. Lorkiewicz P, Riggs DW, Keith RJ, Conklin DJ, Xie Z, Sutaria S, et al. Comparison of Urinary Biomarkers of Exposure in Humans Using Electronic Cigarettes, Combustible Cigarettes, and Smokeless Tobacco. Nicotine Tob Res. 2019;21(9):1228–38.

23. Göney G, Çok İ, Tamer U, Burgaz S, Şengezer T. Urinary cotinine levels of electronic cigarette (e-cigarette) users. Toxicol Mech Methods. 2016;26(6):414–8.

24. Park MB, Choi JK. Differences between the effects of conventional cigarettes, e-cigarettes and

For peer review only

For peer review only - http://bmjopen.bmj.com/site/about/guidelines.xhtml

dual product use on urine cotinine levels. Tob Induc Dis. 2019;17(February):1–9.

25. Al-Houqani M, Leinberger-Jabari A, Al naeemi A, Al Junaibi A, Al Zaabi E, Oumeziane N, et al. Patterns of tobacco use in the United Arab Emirates Healthy Future (UAEHFS) pilot study. PLoS One. 2018;13(5).

26. Hoseini M, Yunesian M, Nabizadeh R, Yaghmaeian K, Parmy S, Gharibi H, et al. Biomonitoring of tobacco smoke exposure and self-reported smoking status among general population of Tehran, Iran. Environ Sci Pollut Res [Internet]. 2016;23(24):25065–73. Available from: http://dx.doi.org/10.1007/s11356-016-7619-8

27. Pearce MS, Mann KD, Singh G, Davison B, Sayers SM. Prevalence and validity of self-reported smoking in Indigenous and non-Indigenous young adults in the Australian Northern Territory. BMC Public Health. 2014;14(1):1–7.

28. Wong SL, Shields M, Leatherdale S, Malaison E, Hammond D, Wong SL, et al. Assessment of validity of self-reported smoking status. Heal Reports. 2012;23(1).

29. Man CN, Fathelrahman AI, Harn GL, Lajis R, Samin ASM, Omar M, et al. Correlation between urinary nicotine, cotinine and self-reported smoking status among educated young adults. Environ Toxicol Pharmacol. 2009;28(1):92–6.

30. Wagenknecht LE, Burke GL, Perkins LL, Haley NJ, Friedman GD. Misclassification of smoking status in the CARDIA study: A comparison of self-report with serum cotinine levels. Am J Public Health. 1992;82(1):33–6.

31. Post A, Gilljam H, Rosendahl I, Meurling L, Bremberg S, Galanti MR. Validity of self reports in a cohort of Swedish adolescent smokers and smokeless tobacco (snus) users. Tob Control. 2005;14(2):114–7.

32. Boykan R, Messina CR, Chateau G, Eliscu A, Tolentino J, Goniewicz ML. Self-Reported Use of Tobacco, E-cigarettes, and Marijuana Versus Urinary Biomarkers. Pediatrics [Internet]. 2019 [cited 2021 Oct 26];143(5):e20183531. Available from:

For peer review only - http://bmjopen.bmj.com/site/about/guidelines.xhtml

https://doi.org/10.1542/peds.2018-3531

33. Williams J, Rakovac I, Loyola E, Sturua L, Maglakelidze N, Gamkrelidze A, et al. A comparison of self-reported to cotinine-detected smoking status among adults in Georgia. Eur J Public Health. 2020;30(5):1007–12.

34. Hwang JH, Kim JY, Lee DH, Jung HG, Park SW. Underestimation of self-reported smoking prevalence in Korean adolescents: Evidence from gold standard by combined method. Int J Environ Res Public Health. 2018;15(4):1–10.

35. Valladolid-López MDC, Barrientos-Gutiérrez T, Reynales-Shigematsu LM, Thrasher JF, Peláez-Ballestas I, Lazcano-Ponce E, et al. Evaluating the validity of self-reported smoking in Mexican adolescents. BMJ Open. 2015;5(10):1–8.

36. Guitérrez-Torres DS, Wang L, Blount BC, Xia B, Sosnoff CS, Shiels MS, et al. Concentrations of Cotinine and 4-(Methylnitrosamino)-1-(3-Pyridyl)-1-Butanol (NNAL) in U.S. Non-Daily Cigarette Smokers. Cancer Epidemiol Biomarkers Prev. 2021;30:1165–74.

37. Hilberink SR, Jacobs JE, van Opstal S, van der Weijden T, Keegstra J, Kempers PLJ, et al. Validation of smoking cessation self-reported by patients with chronic obstructive pulmonary disease. Int J Gen Med. 2011;4:85–90.

38. Alberg AJ, Worley ML, Tooze JA, Hatcher JL, Carpenter MJ, Day TA, et al. The validity of self-reported recent smoking in head and neck cancer surgical patients. Otolaryngol - Head Neck Surg (United States). 2015;153(6):990–5.

39. Gritz ER, Carr CR, Rapkin D, Abemayor E, Chang LJC, Wong WK, et al. Predictors of Long-Term Smoking Cessation in Head and Neck Cancer Patients. Cancer Epidemiol Biomarkers Prev. 1993;2(3):261–70.

40. Ebner N, Földes G, Szabo T, Tacke M, Fülster S, Sandek A, et al. Assessment of serum cotinine in patients with chronic heart failure: Self-reported versus objective smoking behaviour. Clin Res Cardiol. 2013;102(2):95–101.

For peer review only

For peer review only - http://bmjopen.bmj.com/site/about/guidelines.xhtml

41. Wilson DK, Wallston KA, King JE, Smith MS, Heim C. Validation of smoking abstinence in newly diagnosed cardiovascular patients. Addict Behav. 1993;18(4):421–9.

42. England LJ, Grauman A, Qian C, Wilkins DG, Schisterman EF, Yu KF, et al. Misclassification of maternal smoking status and its effects on an epidemiologic study of pregnancy outcomes. Nicotine Tob Res. 2007;9(10):1005–13.

43. Ashford K, Wiggins A, Rayens E, Assef S, Fallin A, Rayens MK. Perinatal biochemical confirmation of smoking status by trimester. Nicotine Tob Res. 2017;19(5):631–5.

44. Haufroid V, Lison D. Urinary cotinine as a tobacco-smoke exposure index. A minireview. Int Arch Occup Environ Health. 1998;71(3):162–8.

45. Davis RA, Stiles MF, deBethizy JD, Reynolds JH. Dietary nicotine: A source of urinary cotinine. Food Chem Toxicol. 1991;29(12):821–7.

46. European Commission. Revision of the Tobacco Products Directive [Internet]. Vol. 2014. 2014. Available from: https://ec.europa.eu/health//sites/health/files/tobacco/docs/dir_201440_en.pdf%0Ahttps://ec.europa.eu/health/sites/health/files/tobacco/docs/dir_201440_en.pdf%0Ahttps://ec.europa.eu/health//sites/health/files/tobacco/docs/dir_201440_en.pdf%0Ahttp://ec.eur

47. Federal Trade Commission. Federal Trade Commission Smokeless Tobacco Report for 2020 [Internet]. 2021. Available from: https://www.ftc.gov/system/files/documents/reports/federal-trade-commission-cigarette-report-2020-smokeless-tobacco-report-2020/p114508fy20smokelesstobacco.pdf

For peer review only - http://bmjopen.bmj.com/site/about/guidelines.xhtml

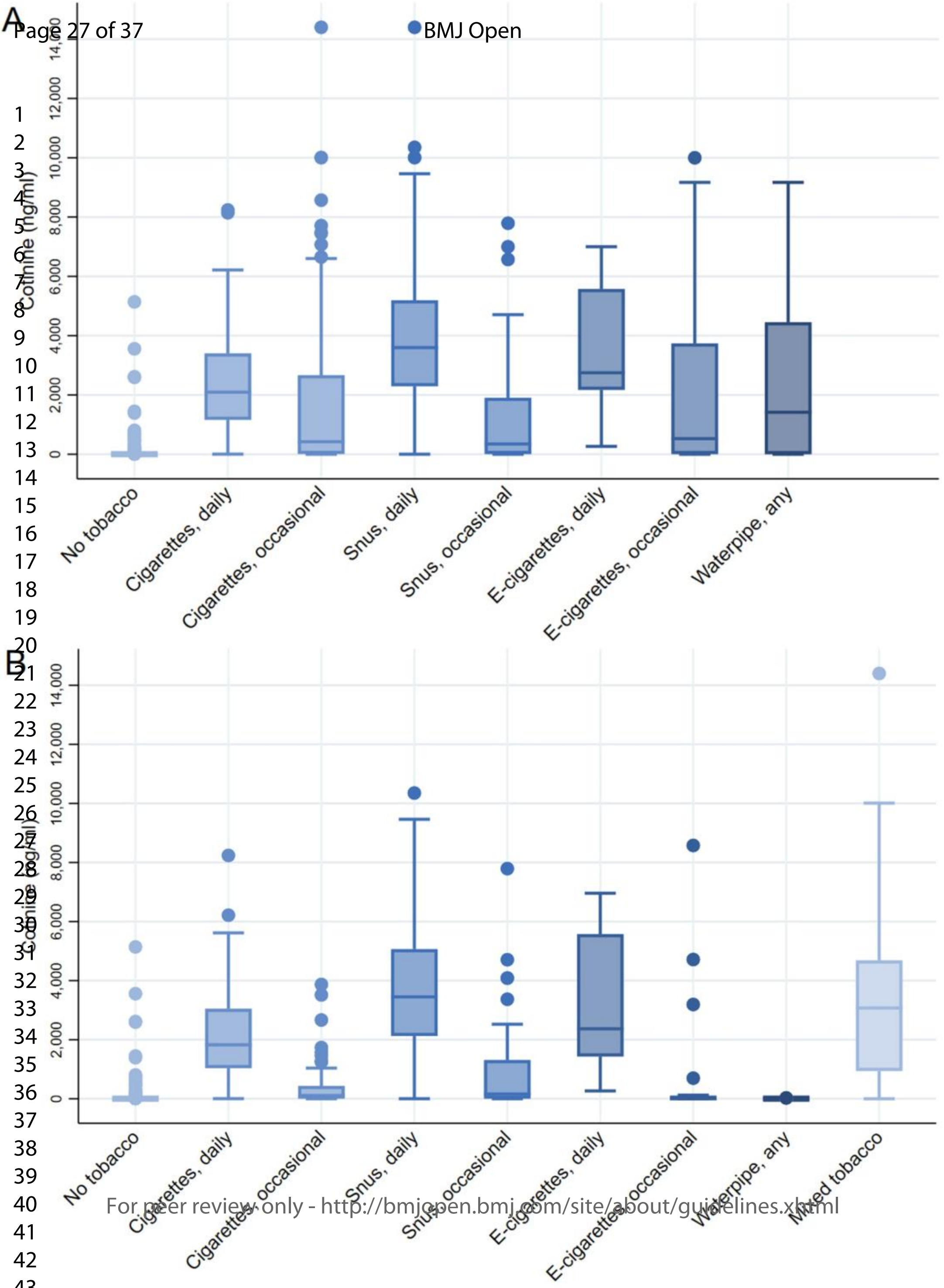
A
Cotinine (ng/ml)
0
2,000
4,000
6,000
8,000
10,000
12,000
14,000
No tobacco
Cigarettes, daily
Cigarettes, occasional
Snus, daily
Snus, occasional
E-cigarettes, daily
E-cigarettes, occasional
Waterpipe, any
B
Cotinine (ng/ml)
0
2,000
4,000
6,000
8,000
10,000
12,000
14,000
No tobacco
Cigarettes, daily
Cigarettes, occasional
Snus, daily
Snus, occasional
E-cigarettes, daily
E-cigarettes, occasional
Waterpipe, any
Mixed tobacco

Validation of self-reported tobacco use among Swedish young adults

Supplementary information

Anna Zettergren, Shanzina Iasmin Sompa, Lena Palmberg, Petter Ljungman, Göran Pershagen, Niklas Andersson, Christian H Lindh, Antonios Georgelis, Inger Kull, Erik Melén, Sandra Ekström, Anna Bergström

For peer review only

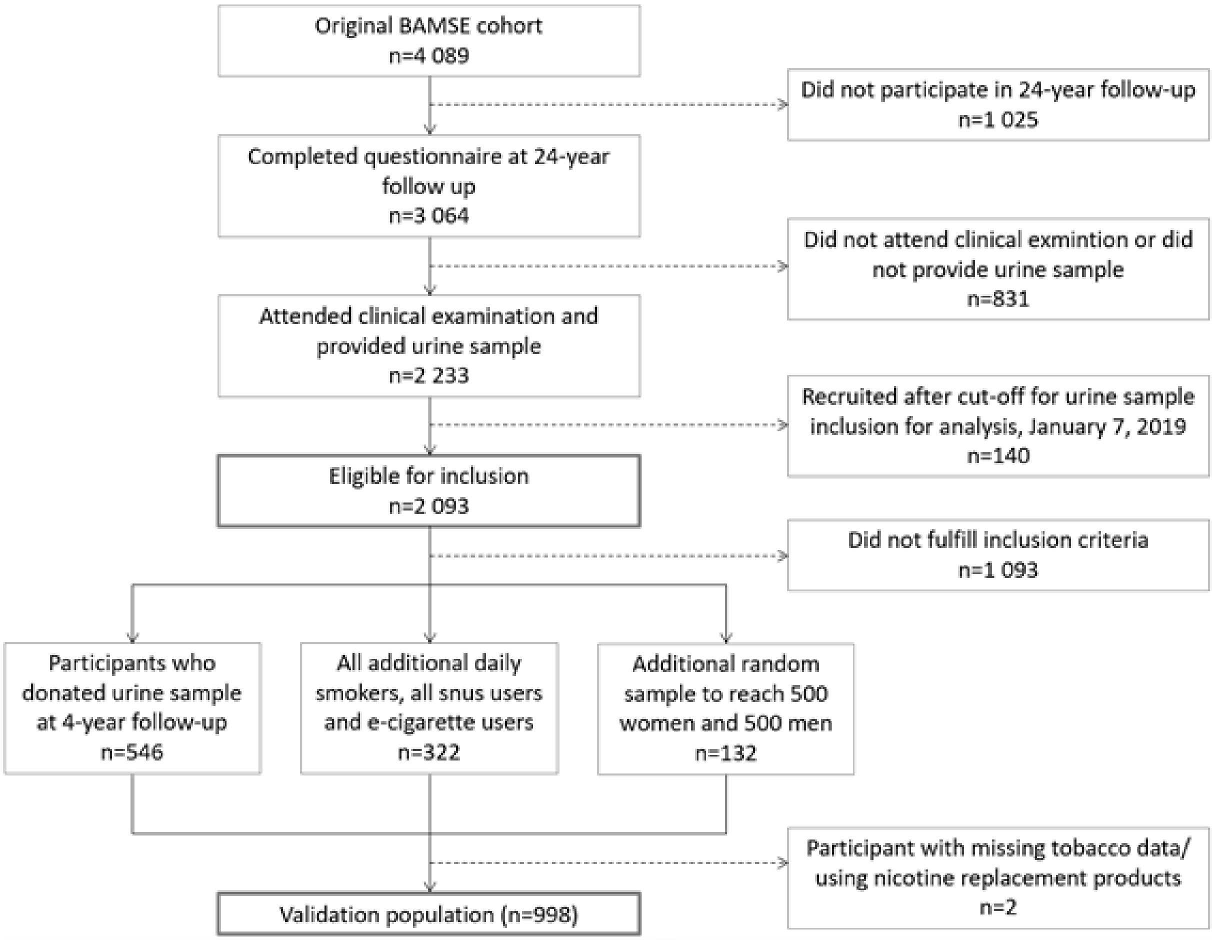

Figure S1. Overview of the selection process of the validation population for urine sample analysis of cotinine.

Variable definitions:

Age: Age in years at questionnaire, as calculated from date of birth.

Socioeconomic status at baseline: Household economic status at enrollment in the BAMSE study. Categorized as i) blue collar worker or ii) white collar worker, based on the highest employment category of the parents.

Any parent smoking at baseline: Any parent smoking at baseline of the BAMSE cohort. Based on self-reported questionnaire data from parents.

Education level: Participants highest completed education level at the time of the 24-year follow-up questionnaire. Categorized as i) elementary school or high school and ii) university or higher.

Occupation: Based on questionnaire data from 24-year follow-up. Categorized as i) student, ii) working or iii) other.

Body mass index (BMI): Calculated as kg/m^2 from weight and height measurements from the 24-year follow-up clinical examination.

Secondhand smoke exposure: Based on self-reported exposure from the 24-year follow-up questionnaire. Categorized as i) "yes" if daily exposed to indoor tobacco smoke (at home, work or other places), otherwise as ii) "no".

Asthma: Defined based on information from the 24-year follow-up questionnaire as "yes" if participants had a doctor's diagnosis of Asthma together with either symptoms of breathing difficulties in the last 12 months OR taking asthma medication in the past 12 months.

For peer review only

Table S1. Self-reported tobacco use in the validation population (n=998).

	All n=998	Women n=499	Men n=499	p-value[a] (women vs. men)
		n (%)		
**Cigarettes**				
Never	588 (59.0)	279 (56.0)	309 (61.9)	0.054
Former	135 (13.5)	60 (12.0)	75 (15.0)	0.165
Occasional	125 (12.5)	64 (12.8)	61 (12.2)	0.774
Daily	150 (15.0)	96 (19.2)	54 (10.8)	<0.0001
<10 cigarettes/day	*82 (55.4)*	*57 (60.0)*	*25 (47.2)*	*0.132*
≥10 cigarettes/day	*66 (44.6)*	*38 (40.0)*	*28 (52.8)*	
**Snus**				
Never	704 (70.5)	429 (86.0)	275 (55.1)	<0.0001
Former	37 (3.7)	5 (1.0)	32 (6.4)	<0.0001
Occasional	75 (7.5)	28 (5.6)	47 (9.4)	0.023
Daily	182 (18.2)	37 (7.4)	145 (29.1)	<0.0001
<4 boxes of snus/week	*99 (54.4)*	*27 (73.0)*	*72 (49.7)*	*0.011*
≥4 boxes of snus/week	*83 (45.6)*	*10 (27.0)*	*73 (50.3)*	
**E-cigarettes**				
Never	918 (92.0)	472 (94.6)	446 (89.4)	0.002
Occasional	71 (7.1)	24 (4.8)	47 (9.4)	0.005
Daily	9 (0.9)	3 (0.6)	6 (1.2)	0.315
**Waterpipe**				
Never	970 (97.4)	488 (97.8)	482 (97.0)	0.421
Occasional	25 (2.5)	10 (2.0)	15 (3.0)	0.306
Daily	1 (0.1)	1 (0.2)	0 (0)	0.318
Number of tobacco types used				
1	393 (77.4)	190 (84.1)	203 (72.0)	0.001
>1	115 (22.6)	36 (15.9)	79 (28.0)	
Secondhand smoke exposure				
Yes[1]	39 (4.0)	24 (4.9)	15 (3.1)	0.141

[a]: P-value from Pearson χ^2 test.
[b]: P-value from two-tailed t-test.
[c]: P-value from Wilcoxon rank-sum test
[1]: Defined as daily exposure to indoor tobacco smoke.

Table S2. Urinary cotinine levels (ng/ml) by tobacco use categories, adjusted for specific gravity.

	n	Median	Mean	SD	95th percentile	Min-max
**Cigarettes**						
Any cigarette smoking	275	1 465	2 089	2 176	6 215	0.1–14 399
Any cigarette smoking, mixed users excluded	*176*	*1 180*	*1 494*	*1 545*	*4 528*	*0.1–8 238*
Daily cigarette smoking	150	2 094	2 390	1 670	5 264	3.6–8 238
Daily cigarette smoking, mixed users excluded	*112*	*1 826*	*2 101*	*1 547*	*4 957*	*3.6–8 238*
Occasional cigarette smoking	125	422	1 728	2 621	7 449	0.1–14 399
Occasional cigarette smoking, mixed users excluded	*64*	*109*	*433*	*787*	*1 733*	*0.1–3 868*
**Snus**						
Any snus use	257	2 939	3 159	2 510	7 790	0.3–14 399
Any snus use, mixed users excluded	*174*	*2 566*	*2 834*	*2 368*	*7 381*	*0.3–10 350*
Daily snus use	182	3 599	3 950	2 346	8 051	1.7–14 399
Daily snus use, mixed users excluded	*119*	*3 449*	*3 726*	*2 125*	*7 859*	*1.7–10 350*
Occasional snus use	75	345	1 239	1 752	4 707	0.3–7 790
Occasional snus use, mixed users excluded	*55*	*161*	*903*	*1 500*	*4 088*	*0.3–7 790*
**E-cigarettes**						
Any e-cigarette smoking	80	874	2 159	2 697	8 359	0.3–9 999
Any e-cigarette smoking, mixed users excluded	*32*	*10.7*	*1 219*	*2 260*	*6 958*	*0.5–8 574*
Daily e-cigarette smoking	9	2 749	3 643	2 431	6 998	265–6 998
Daily e-cigarette smoking, mixed users excluded	*7*	*2 368*	*3 074*	*2 357*	*6 958*	*265–6 958*
Occasional e-cigarette smoking	71	527	1 971	2 686	8 574	0.3–9 999
Occasional e-cigarette smoking, mixed users excluded	*25*	*5.7*	*700*	*1 981*	*4 714*	*0.5–8 574*
**Waterpipe**						
Any waterpipe use	26	1 415	2 400	2 698	6 602	0.3–9 169
Any waterpipe use, mixed users excluded	*9*	*2.0*	*7.2*	*10.1*	*28.9*	*0.7–28.9*

For peer review only

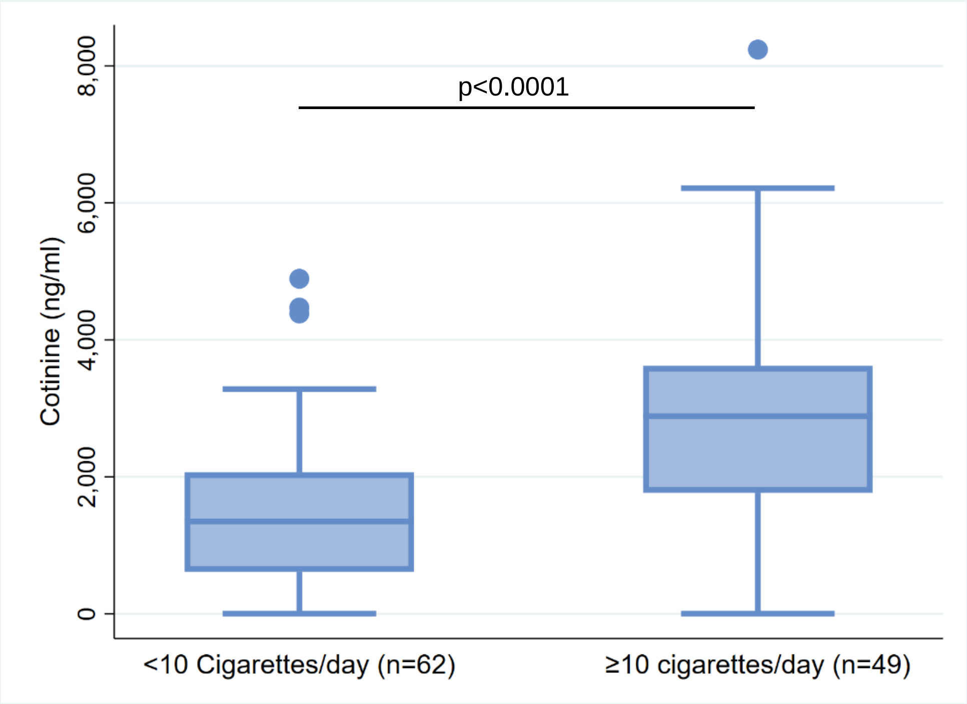

Figure S2. Urinary cotinine levels among daily cigarette smokers, by number of cigarettes per day. Participants with mixed tobacco use excluded.

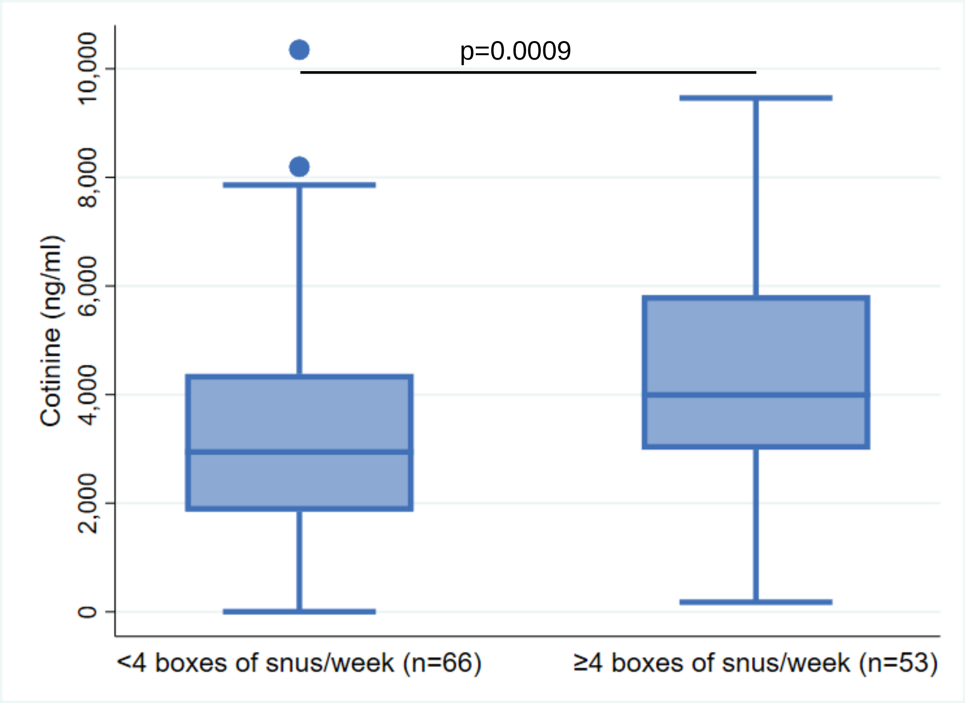

Figure S3. Urinary cotinine levels among daily snus users, by number of boxes snus per week. Participants with mixed tobacco use excluded.

Table S3. Self-reported tobacco use against urinary cotinine levels by type of tobacco and frequency of use, stratified by gender.

			Women	Men
Cigarettes	Daily	κ:	0.838	0.801
		Sensitivity:	83.3%	75.4%
		Specificity:	97.7%	99.0%
		PVV:	93.8%	96.3%
		NPV:	93.4%	92.2%
	Occasional	κ:	0.592	0.643
		Sensitivity:	69.5%	72.1%
		Specificity:	91.7%	92.2%
		PVV:	64.1%	72.1%
		NPV:	93.4%	92.2%
	Any	κ:	0.764	0.760
		Sensitivity:	87.9%	85.0%
		Specificity:	89.8%	91.3%
		PVV:	81.9%	83.5%
		NPV:	93.4%	92.2%
Snus	Daily	κ:	0.726	0.898
		Sensitivity:	65.4%	89.4%
		Specificity:	98.8%	99.5%
		PVV:	91.9%	99.3%
		NPV:	93.4%	92.2%
	Occasional	κ:	0.461	0.640
		Sensitivity:	47.1%	67.3%
		Specificity:	95.5%	94.3%
		PVV:	57.1%	74.5%
		NPV:	93.4%	92.2%
	Any	κ:	0.691	0.853
		Sensitivity:	73.5%	91.3%
		Specificity:	94.4%	93.9%
		PVV:	76.9%	93.2%
		NPV:	93.4%	92.2%
E-cigarette use	Any	κ:	0.418	0.658
		Sensitivity:	43.8%	70.2%
		Specificity:	95.1%	93.9%
		PVV:	51.9%	75.5%
		NPV:	93.4%	92.2%

Cigarette smoking: Analysis including all self-reported cigarette smokers and all non-tobacco users.
Snus use: Analysis including all self-reported snus users and all non-tobacco users.
E-cigarette use: Analysis including all self-reported E-cigarette users and all non-tobacco users.
Daily: Results from analyses excluding occasional tobacco users.
Occasional: Results from analyses excluding daily tobacco users.
Any: Results from analyses combining daily and occasional tobacco users.
κ: Cohen's kappa coefficient.
PPV: Positive predictive value
NPV: Negative predictive value

For peer review only

Table S4. Self-reported tobacco use against urinary cotinine levels, excluding participants with mixed tobacco use or with missing data on waterpipe use (n=117 excluded).

Self-reports	Urinary cotinine level						
**Cigarette smoking**					**Daily**	**Occasional**	**Any**
	<50 ng/ml	≥50 ng/ml	*Total*				
No	455	35	*490*	κ:	0.784	0.469	0.725
Occasional	28	36	*64*	Sensitivity:	74.8%	50.7%	80.0%
Daily	8	104	*112*	Specificity:	98.3%	94.2%	92.7%
Total	*491*	*175*	*666*	PPV:	92.9%	56.3%	79.5%
				NPV:	92.9%	92.9%	92.9%
**Snus use**					**Daily**	**Occasional**	**Any**
	<50 ng/ml	≥50 ng/ml	*Total*				
No	455	35	*490*	κ:	0.825	0.478	0.775
Occasional	22	33	*55*	Sensitivity:	77.0%	48.5%	81.1%
Daily	2	117	*119*	Specificity:	99.6%	95.4%	95.0%
Total	*479*	*185*	*664*	PPV:	98.3%	60.0%	86.2%
				NPV:	92.9%	92.9%	92.9%
**E-cigarette use**					**Any**		
	<50 ng/ml	≥50 ng/ml	*Total*				
No	455	35	*490*	κ:	0.293		
Occasional	18	7	*25*	Sensitivity:	28.6%		
Daily	0	7	*7*	Specificity:	96.2%		
Total	*473*	*49*	*522*	PPV:	43.8%		
				NPV:	92.9%		

Cigarette smoking: Analysis including self-reported cigarette smokers without other tobacco use and all non-tobacco users.
Snus use: Analysis including self-reported snus users without other tobacco use and all non-tobacco users.
E-cigarette use: Analysis including self-reported e-cigarette users without other tobacco use and all non-tobacco users.
Daily: Results from analyses excluding occasional tobacco users.
Occasional: Results from analyses excluding daily tobacco users.
Any: Results from analyses combining daily and occasional tobacco users.
κ: Cohen's kappa coefficient.
PPV: Positive predictive value
NPV: Negative predictive value

For peer review only

Table S5. Self-reported tobacco use against urinary cotinine levels for participants who provided urine samples within 6 weeks after answering the questionnaire (n=507 included).

Self-reports	Urinary cotinine level						
**Cigarette smoking**					**Daily**	**Occasional**	**Any**
	<50 ng/ml	≥50 ng/ml	*Total*				
No	216	15	*231*	κ:	0.867	0.639	0.785
Occasional	22	47	*69*	Sensitivity:	84.4%	75.8%	89.5%
Daily	2	81	*83*	Specificity:	99.1%	90.8%	90.0%
Total	*240*	*143*	*383*	PPV:	97.6%	68.1%	84.2%
				NPV:	93.5%	93.5%	93.5%
**Snus use**					**Daily**	**Occasional**	**Any**
	<50 ng/ml	≥50 ng/ml	*Total*				
No	216	15	*231*	κ:	0.890	0.643	0.861
Occasional	8	26	*34*	Sensitivity:	87.0%	63.4%	89.4%
Daily	1	101	*102*	Specificity:	99.5%	96.4%	96.0%
Total	*225*	*142*	*367*	PPV:	99.0%	76.5%	93.4%
				NPV:	93.5%	93.5%	93.5%
**E-cigarette use**					**Any**		
	<50 ng/ml	≥50 ng/ml	*Total*				
No	216	15	*231*	κ:	0.602		
Occasional	15	25	*40*	Sensitivity:	66.7%		
Daily	0	5	*5*	Specificity:	93.5%		
Total	*231*	*45*	*276*	PPV:	66.7%		
				NPV:	93.5%		

Cigarette smoking: Analysis including all self-reported cigarette smokers and all non-tobacco users.
Snus use: Analysis including all self-reported snus users and all non-tobacco users.
E-cigarette use: Analysis including all self-reported E-cigarette users and all non-tobacco users.
Daily: Results from analyses excluding occasional tobacco users.
Occasional: Results from analyses excluding daily tobacco users.
Any: Results from analyses combining daily and occasional tobacco users.
κ: Cohen's kappa coefficient.
PPV: Positive predictive value
NPV: Negative predictive value

For peer review only

STROBE 2007 (v4) Statement—Checklist of items that should be included in reports of *cross-sectional studies*

Section/Topic	Item #	Recommendation	Reported on page #
**Title and abstract**	1	(*a*) Indicate the study's design with a commonly used term in the title or the abstract	1
		(*b*) Provide in the abstract an informative and balanced summary of what was done and what was found	2
**Introduction**			
Background/rationale	2	Explain the scientific background and rationale for the investigation being reported	4-5
Objectives	3	State specific objectives, including any prespecified hypotheses	5
**Methods**			
Study design	4	Present key elements of study design early in the paper	5-6
Setting	5	Describe the setting, locations, and relevant dates, including periods of recruitment, exposure, follow-up, and data collection	5-6
Participants	6	(*a*) Give the eligibility criteria, and the sources and methods of selection of participants	5-6
Variables	7	Clearly define all outcomes, exposures, predictors, potential confounders, and effect modifiers. Give diagnostic criteria, if applicable	7-8
Data sources/ measurement	8*	For each variable of interest, give sources of data and details of methods of assessment (measurement). Describe comparability of assessment methods if there is more than one group	7-8
Bias	9	Describe any efforts to address potential sources of bias	7-8
Study size	10	Explain how the study size was arrived at	5-6
Quantitative variables	11	Explain how quantitative variables were handled in the analyses. If applicable, describe which groupings were chosen and why	7-8
Statistical methods	12	(*a*) Describe all statistical methods, including those used to control for confounding	7-8
		(*b*) Describe any methods used to examine subgroups and interactions	7-8
		(*c*) Explain how missing data were addressed	8
		(*d*) If applicable, describe analytical methods taking account of sampling strategy	
		(*e*) Describe any sensitivity analyses	7
**Results**			

For peer review only

Participants	13*	(a) Report numbers of individuals at each stage of study—eg numbers potentially eligible, examined for eligibility, confirmed eligible, included in the study, completing follow-up, and analysed	5-6
		(b) Give reasons for non-participation at each stage	5-6
		(c) Consider use of a flow diagram	2 in supplement
Descriptive data	14*	(a) Give characteristics of study participants (eg demographic, clinical, social) and information on exposures and potential confounders	8-11
		(b) Indicate number of participants with missing data for each variable of interest	Table 1
Outcome data	15*	Report numbers of outcome events or summary measures	11-12
Main results	16	(*a*) Give unadjusted estimates and, if applicable, confounder-adjusted estimates and their precision (eg, 95% confidence interval). Make clear which confounders were adjusted for and why they were included	12-14
		(*b*) Report category boundaries when continuous variables were categorized	12-14
		(*c*) If relevant, consider translating estimates of relative risk into absolute risk for a meaningful time period	
Other analyses	17	Report other analyses done—eg analyses of subgroups and interactions, and sensitivity analyses	12
**Discussion**			
Key results	18	Summarise key results with reference to study objectives	14
Limitations	19	Discuss limitations of the study, taking into account sources of potential bias or imprecision. Discuss both direction and magnitude of any potential bias	14 & 17
Interpretation	20	Give a cautious overall interpretation of results considering objectives, limitations, multiplicity of analyses, results from similar studies, and other relevant evidence	14-17
Generalisability	21	Discuss the generalisability (external validity) of the study results	17
**Other information**			
Funding	22	Give the source of funding and the role of the funders for the present study and, if applicable, for the original study on which the present article is based	19

*Give information separately for cases and controls in case-control studies and, if applicable, for exposed and unexposed groups in cohort and cross-sectional studies.

Note: An Explanation and Elaboration article discusses each checklist item and gives methodological background and published examples of transparent reporting. The STROBE checklist is best used in conjunction with this article (freely available on the Web sites of PLoS Medicine at http://www.plosmedicine.org/, Annals of Internal Medicine at http://www.annals.org/, and Epidemiology at http://www.epidem.com/). Information on the STROBE Initiative is available at www.strobe-statement.org.

For peer review only

BMJ Open

Assessing tobacco use in Swedish young adults from self-report and urinary cotinine: a validation study using the BAMSE birth cohort

Journal:	*BMJ Open*
Manuscript ID	bmjopen-2023-072582.R1
Article Type:	Original research
Date Submitted by the Author:	16-Jun-2023
Complete List of Authors:	Zettergren, Anna; Karolinska Institutet, Institute of Environmental Medicine<br>Sompa, Shanzina; Karolinska Institutet, Institute of Environmental Medicine<br>Palmberg, Lena; Karolinska Institutet, Institute of Environmental Medicine<br>Ljungman, Petter; Karolinska Institutet, Institute of Environmental Medicine; Danderyd University Hospital, Department of Cardiology<br>Pershagen, Göran; Karolinska Institutet, Institute of Environmental Medicine<br>Andersson, Niklas; Institute of Environmental Medicine, Karolinska Institutet<br>Lindh, Christian ; Lund University, Division of Occupational and Environmental Medicine<br>Georgelis, Antonios; Karolinska Institutet, Institute of Environmental Medicine; Region Stockholm, Centre for Occupational and Environmental Medicine<br>Kull, Inger; Södersjukhuset AB, Sachs' Children and Youth Hospital; Karolinska Institutet, Department of Clinical Science and Education, Södersjukhuset<br>Melen, Erik; Södersjukhuset AB, Sachs' Children and Youth Hospital; Karolinska Institutet, Department of Clinical Science and Education, Södersjukhuset<br>Ekström, Sandra; Karolinska Institute; Region Stockholm, Centre for Occupational and Environmental Medicine<br>Bergstrom, Anna; Karolinska Institutet, Institute of Environmental Medicine
**Primary Subject Heading**:	Smoking and tobacco
Secondary Subject Heading:	Epidemiology, Public health
Keywords:	EPIDEMIOLOGY, PUBLIC HEALTH, STATISTICS & RESEARCH METHODS

SCHOLARONE™
Manuscripts

I, the Submitting Author has the right to grant and does grant on behalf of all authors of the Work (as defined in the below author licence), an exclusive licence and/or a non-exclusive licence for contributions from authors who are: i) UK Crown employees; ii) where BMJ has agreed a CC-BY licence shall apply, and/or iii) in accordance with the terms applicable for US Federal Government officers or employees acting as part of their official duties; on a worldwide, perpetual, irrevocable, royalty-free basis to BMJ Publishing Group Ltd ("BMJ") its licensees and where the relevant Journal is co-owned by BMJ to the co-owners of the Journal, to publish the Work in this journal and any other BMJ products and to exploit all rights, as set out in our licence.

The Submitting Author accepts and understands that any supply made under these terms is made by BMJ to the Submitting Author unless you are acting as an employee on behalf of your employer or a postgraduate student of an affiliated institution which is paying any applicable article publishing charge ("APC") for Open Access articles. Where the Submitting Author wishes to make the Work available on an Open Access basis (and intends to pay the relevant APC), the terms of reuse of such Open Access shall be governed by a Creative Commons licence – details of these licences and which Creative Commons licence will apply to this Work are set out in our licence referred to above.

Other than as permitted in any relevant BMJ Author's Self Archiving Policies, I confirm this Work has not been accepted for publication elsewhere, is not being considered for publication elsewhere and does not duplicate material already published. I confirm all authors consent to publication of this Work and authorise the granting of this licence.

For peer review only

Assessing tobacco use in Swedish young adults from self-report and urinary cotinine: a validation study using the BAMSE birth cohort

Anna Zettergren[1,*], Shanzina Iasmin Sompa[1], Lena Palmberg[1], Petter Ljungman[1,2], Göran Pershagen[1], Niklas Andersson[1], Christian H Lindh[3], Antonios Georgelis[1,4], Inger Kull[5,6], Erik Melén[1,5,6], Sandra Ekström[1,4], Anna Bergström[1,4]

1. Institute of Environmental Medicine, Karolinska Institutet, Stockholm Sweden

2. Department of Cardiology, Danderyd Hospital, Stockholm Sweden.

3. Division of Occupational and Environmental Medicine, Lund University, Lund, Sweden

4. Centre for Occupational and Environmental Medicine, Region Stockholm, Stockholm, Sweden

5. Sachs' Children and Youth Hospital, Södersjukhuset, Stockholm, Sweden

6. Department of Clinical Science and Education, Södersjukhuset, Karolinska Institutet, Stockholm, Sweden

*** Corresponding author:**

Anna Zettergren

Institute of Environmental Medicine, Karolinska Institutet, Stockholm Sweden.

E-mail: anna.zettergren@ki.se

Telephone: +46702022024

Word count: 4107

For peer review only - http://bmjopen.bmj.com/site/about/guidelines.xhtml

ABSTRACT

Objectives: Studies on health effects of tobacco often rely on self-reported exposure data, which is subjective and can lead to misclassification. The aim of this study was to describe the prevalence of cigarette smoking, snus and e-cigarette use, as well as to validate self-reported tobacco use among young adults in Sweden.

Method: Participants of a population-based Swedish cohort (n=3 052), aged 22-25 years, assessed their tobacco use in a web-questionnaire. Urinary cotinine was analyzed in a subsample of the study population (n=998). The agreement between self-reported tobacco use and urinary cotinine was assessed using Cohen's Kappa coefficient (κ) at a cut-off level of 50 ng/ml.

Results: Patterns of tobacco use differed between men and women. Among men, 20.0% reported daily snus use, 5.8% daily cigarette smoking and 5.6% any e-cigarette use. In contrast, 3.2% of the women reported daily snus use, 9.0% daily cigarette smoking and 2.4% any e-cigarette use. Among the tobacco use categories, daily snus users had the highest levels of cotinine. Of reported non-tobacco users, 3.5% had cotinine levels above the cut-off, compared to 68.0% among both occasional cigarette smokers and snus users, 67.5% among all e-cigarette users and 94.7% and 97.8% among daily cigarette smokers and snus users, respectively. Agreement between self-reported tobacco use and urinary cotinine was classified as strong for daily use of cigarettes (κ =0.824) and snus (κ=0.861), while moderate to weak for occasional smoking (κ=0.618), occasional snus use (κ=0.573) and any e-cigarette use (κ=0.576).

Conclusions: We found high validity of self-reported tobacco use in our study population, particularly for daily tobacco use. Further we found that daily snus users were exposed to high levels of cotinine. Together with previous findings, our results indicate good validity of self-reported tobacco use among young adults.

For peer review only - http://bmjopen.bmj.com/site/about/guidelines.xhtml

STRENGTH AND LIMITATIONS OF THIS STUDY

- Several types of tobacco use were validated using biochemical verification.
- This is the first study to validate snus use in a population of young adults comprised of both men and women.
- A large study sample including both young men and women was analyzed.
- The study was conducted within a well characterized, population-based birth cohort.
- A time lag existed between reporting tobacco use and biochemical verification.

For peer review only - http://bmjopen.bmj.com/site/about/guidelines.xhtml

INTRODUCTION

Research on health effects of tobacco mainly relies on self-reported exposure data. This type of exposure assessment is subjective and may result in under- or overreporting(1). Objective assessment of tobacco use can be performed using cotinine, a metabolite of nicotine, as a biomarker in urine, blood or saliva(2). However, these methods are expensive and time consuming, and are therefore not feasible for large populations. Instead, biomarker data can be used to validate self-reported data in smaller subpopulations, to estimate validity in larger cohorts.

Self-reported cigarette smoking has been validated in several studies with varying results in different populations, but with generally high validity(1). However, for other types of tobacco and nicotine products, such as smokeless tobacco and electronic cigarettes (e-cigarettes), validation studies are scarce(3). In Sweden, the smokeless tobacco type snus is used to a higher extent than cigarettes(4). While cigarette smoking has decreased in the Swedish adult population over the last several decades, to 6% daily smokers in 2022, daily snus consumption rates have increased to a current 14%, with the largest increases being among younger populations(4). Men have a notably higher consumption of snus compared to women in Sweden, however women's consumption is increasing rapidly. Although snus share some active compounds with cigarettes, much less is known about its health effects, particularly among young adults(5). Self-reported snus use has been validated among Swedish pregnant women, where high agreement between self-reported snus use and urinary cotinine levels was found(6). Still, no study has investigated the validity of self-reported information on snus use among young men and non-pregnant women.

E-cigarette use has increased globally the past decade, especially among young populations. In Sweden, an estimated 2% of the population use e-cigarettes, with the highest prevalence among men under 30 years(4,7). Although initially intended to reduce the harmful effects of cigarettes by facilitating smoking cessation, e-cigarettes are currently under debate as to whether they rather cause adverse health effects among adolescents and young adults(8). Still, more research is needed to evaluate potential health effects of e-cigarettes(9).

For peer review only - http://bmjopen.bmj.com/site/about/guidelines.xhtml

With the need for research on health effects of tobacco and nicotine products among young adults comes the need for reliable exposure data. Further, as validity of self-reported tobacco use may change over time and be influenced by factors such as age and gender, as well as smoking habits and attitudes towards tobacco products, it is important to regularly perform validation studies in different populations. Therefore, the aims of this study were to describe the prevalence of cigarette smoking, snus use, and e-cigarette use as well as to validate self-reported use of these tobacco types using urinary cotinine among young adults in a Swedish cohort.

METHODS

Study population

This cross-sectional study was conducted within the population-based Swedish birth cohort BAMSE. Detailed information on the BAMSE study is provided elsewhere(10,11). The cohort consists of 4089 newborn children recruited between 1994 and 1996 in Stockholm, Sweden. The participants have been followed up with repeated questionnaires on lifestyle and health up to approximately 24 years of age. On several occasions, participants who answered the questionnaire were invited to clinical examinations. At the follow up at around age 24, hereafter called the 24-year follow-up, conducted between December 2016 and May 2019, the response rate of the questionnaire and the attendance rate at the clinical examination were 75% (n=3 064) and 56% (n=2 270) of the original cohort, respectively.

The study was approved by the Regional Ethics Committee at Karolinska Institutet in Stockholm, Sweden and conducted according to the Helsinki Declaration. All participants provided written informed consent.

Patient and public involvement

Participants or the public were not involved in the design, conduct, reporting, or dissemination plans or the study.

Self-reported tobacco use

For peer review only

During the 24-year follow-up, participants provided self-reported information on tobacco consumption in a web-questionnaire. The current study included all participants who provided information on use of cigarettes, snus, and e-cigarettes (n=3 052), hereafter referred to as the study population. To capture long-term patterns and habitual tobacco use, participants were asked "do you smoke [cigarettes]?" (answer options: "No"; "No, but I used to smoke"; "Yes, sometimes"; "Yes, daily"), "do you use snus?" ("No"; "No, but I used to use snus"; "Yes, sometimes"; "Yes, daily"), "do you use electronic cigarettes?" ("No"; "Yes, sometimes"; "Yes, daily") and "do you smoke waterpipe?" ("No"; "Yes, every month"; "Yes, every week"; "Yes, daily"). For cigarettes and snus, the participants were asked to estimate average consumption per day (for daily smokers), week (for daily snus users and occasional cigarette smokers) or month (for occasional snus users) using open ended questions. Age of regular use debut (at least once per week) of snus or cigarettes was also assessed. In addition, participants were asked whether they had used nicotine replacement products on the day of urine sampling.

Validation population

To validate self-reported tobacco use, a subsample of the BAMSE 24-year follow-up was chosen for urine analysis of cotinine. The selection was conducted among participants who answered the questionnaire and donated a urine sample during the clinical examination at age 24 years. By design, all who reported daily smoking, daily or occasional snus use, daily or occasional e-cigarette use as well as all participants who provided a urine sample at a previous follow-up at age 4 years were included. The selection criteria resulted in 429 men and 439 women. An additional random selection of participants was conducted to reach a subsample of 500 men and 500 women. One participant with missing information on cigarette use and one participant who reported use of nicotine replacement products on the day of urine sampling were excluded, resulting in a validation population size of n=998. See **Figure S1** in the supplement for an overview of the selection process.

Cotinine analysis

For peer review only

For peer review only - http://bmjopen.bmj.com/site/about/guidelines.xhtml

Urine samples collected at the clinical examination during the 24-year follow up were analyzed for cotinine at the Division for Occupational and Environmental Medicine at Lund University. Samples were stored in -80°C until analysis. Urinary cotinine was analyzed using a modified method for serum(12) using liquid chromatography tandem mass spectrometry (LC-MS/MS; QTRAP 5500, Framingham, MA, USA). Briefly, urine samples were prepared in 96-well plates, diluted in buffer and hydrolyzed using β-glucuronidase after deuterium-labelled internal standard was added. Samples were analyzed randomized, and each batch was analyzed with calibration standards, quality controls and chemical blank samples (Milli-Q water). The limit of detection (LOD) for cotinine was set to 1 ng/ml. Specific gravity (SG) was used to compensate for urine sample dilution and was measured using a digital refractometer. The analysis of cotinine is part of a quality control program between analytical laboratories coordinated by the University of Erlangen-Nuremberg, Germany.

Statistical methods

Comparisons of background characteristics and tobacco use between men and women were performed with two-tailed t-tests, Wilcoxon rank-sum test or Pearson χ^2 test, as appropriate. The average consumption of cigarettes was categorized as <10, ≥10 cigarettes per day for daily smokers, based on an *a priori* decided cut-off. Snus consumption was categorized as <4 and ≥4 boxes of snus per week for daily snus users, based on sample distribution. Cotinine levels were adjusted for SG according to: $PSG=P * [(1.014) / (SG-1)]$, where *PSG* is the adjusted cotinine concentration, *P* is the detected cotinine concentration (ng/ml), 1.014 is the average SG in the validation population and *SG* is the specific gravity of the individual urine sample(13). Cotinine levels between tobacco types and gender were compared using Wilcoxon rank-sum test.

To assess the agreement between self-reported tobacco use and urinary cotinine levels, Cohen's Kappa coefficient was calculated at a cut-off level of 50 ng/ml cotinine, as recommended by a subcommittee of the Society for tobacco and nicotine Research(14). Kappa coefficients were interpreted according to McHugh (2012) (15) as 0.00–0.20: no agreement, 0.21–0.39: minimal agreement, 0.40–0.59: weak agreement, 0.60–0.79: moderate agreement, 0.80–0.90: strong agreement, and >0.90: almost perfect agreement. Sensitivity, specificity, positive predictive value (PPV) and negative predictive value

For peer review only - http://bmjopen.bmj.com/site/about/guidelines.xhtml

(NPV) were also estimated. Analyses were stratified by type of tobacco (cigarettes, snus or e-cigarettes), daily or occasional use and gender. The use of waterpipe was not validated due to few users, but users of waterpipe were considered as tobacco users. Sensitivity analyses were performed excluding participants with mixed tobacco use (i.e. using more than one tobacco type) or with missing data on waterpipe use (n=117). Another set of analysis was performed excluding those who provided the urine sample more than 6 weeks after answering the web-questionnaire (n=492).

A prediction model was performed using logistic regression to assess factors associated with false negative reports of tobacco use, so-called underreporting (i.e., reporting no tobacco use but having a urine sample with ≥50 ng/ml cotinine). Factors included in the final model were identified using a stepwise backward selection approach according to Kirkwood & Sterne(16), eliminating factors at a threshold level of $p<0.2$ from a log-likelihood ratio test. Factors considered were identified from the literature and included gender, parental socioeconomic status and parental smoking obtained from the baseline questionnaire; educational level, occupation, asthma, former smoking, former snus use, secondhand smoke exposure, body mass index (BMI), weekday of urine sampling and time between web-questionnaire and urine sampling obtained from the 24-year follow-up. For details on covariate definitions, see supplement. Participants with missing data were excluded from the stepwise backwards selection process, in total affecting 4.8% of participants. The highest rate of missing data was found for second-hand smoke exposure (2.4%), followed by parental socioeconomic status (1.6%).

Statistical analyses were performed in STATA (version 16; Stata Corp, College Station, TX, USA). Statistical significance was considered at $p<0.05$.

RESULTS

Description of study population

An overview of sociodemographic characteristics of the study population can be seen in **Table 1**. The mean age was 22.5 years (range 21.5–25.2 years), 53.0% were women and most participants were students (51.2%), followed by employed workers (40.9%) and other occupations (7.9%). The median

For peer review only - http://bmjopen.bmj.com/site/about/guidelines.xhtml

time between answering the questionnaire and the urine sampling was 41 days (interquartile range 16–84 days).

For peer review only

For peer review only - http://bmjopen.bmj.com/site/about/guidelines.xhtml

Table 1. Sociodemographic characteristics of the study population and the validation population at the BAMSE 24-year follow-up.

	Study population n=3052	Validation population n=998
	**n (%)**	**n (%)**
Male	1 435 (47.0)	499 (50.0)
White collar at baseline	2 537 (84.5)	811 (82.6)
Any parent smoking at baseline	618 (20.4)	209 (21.1)
Asthma	345 (11.3)	137 (13.7)
University degree at age 24	1 093 (36.0)	360 (36.2)
Main occupation:		
Student	1 561 (51.2)	486 (48.7)
Working	1 246 (40.9)	438 (43.9)
Other	240 (7.9)	74 (7.4)
	**Mean (SD)**	**Mean (SD)**
Age, years	22.5 (0.6)	22.4 (0.4)
BMI[a], kg/m^2	23.1 (3.9)	23.4 (4.1)
	**Median (IQR)**	**Median (IQR)**
Time between questionnaire and urine sampling[a]	41 (16-84)	42 (16-78)

Percentages may not add up due to missing values.
[a]: Data available from participants who attended the clinical examination at the 24-year follow-up, 74% (n=2264) of study population
SD: Standard deviation
IQR: Interquartile range

Self-reported tobacco use

Self-reported tobacco use is presented in **Table 2**. Smoking was reported by 20.7% (7.5% daily and 13.3% occasionally), snus use by 15.6% (11.1% daily and 4.5% occasionally) and e-cigarettes by 3.9% (0.5% daily and 3.4% occasionally). Women reported daily cigarette smoking to a larger extent than men, while men reported higher snus and e-cigarette use. A total of 34.3% of the participants reported some type of tobacco use, and among these, 20.8% used more than one type. Among daily smokers, the average number of cigarettes per day was 8.6 among women and 9.7 among men (p=0.085). The average number of boxes per week among daily snus users was significantly higher among men than women (3.0 vs. 1.8, p=0.003). Women began to smoke cigarettes regularly at a younger age than men, while men were younger when beginning to use snus regularly. Secondhand smoke exposure was reported by 3.1% (5.7% among tobacco users and 1.9% among non-users).

For peer review only - http://bmjopen.bmj.com/site/about/guidelines.xhtml

Table 2. Self-reported tobacco use in the BAMSE cohort, at the 24-year follow up.

	All n=3 052	Women n=1 617	Men n=1 435	p-value[a] (women vs. men)
		n (%)		
**Cigarettes**				
Never	2 037 (66.7)	1 056 (65.3)	981 (68.4)	0.074
Former	382 (12.5)	196 (12.1)	186 (13.0)	0.484
Occasional	405 (13.3)	220 (13.6)	185 (12.9)	0.562
Daily	228 (7.5)	145 (9.0)	83 (5.8)	0.001
<10 cigarettes/day	*122 (54.2)*	*84 (58.3)*	*38 (46.9)*	*0.099*
≥10 cigarettes/day	*103 (45.2)*	*60 (41.4)*	*43 (51.8)*	
**Snus**				
Never	2 459 (80.6)	1 481 (91.6)	978 (68.2)	<0.0001
Former	116 (3.8)	27 (1.7)	89 (6.2)	<0.0001
Occasional	138 (4.5)	57 (3.5)	81 (5.6)	0.005
Daily	339 (11.1)	52 (3.2)	287 (20.0)	<0.0001
<4 boxes of snus/week	*184 (54.4)*	*34 (65.4)*	*150 (52.4)*	*0.085*
≥4 boxes of snus/week	*154 (45.6)*	*18 (34.6)*	*136 (47.6)*	
**E-cigarettes**				
No	2 932 (96.1)	1 578 (97.6)	1 354 (94.4)	<0.0001
Occasional	105 (3.4)	35 (2.2)	70 (4.9)	<0.0001
Daily	15 (0.5)	4 (0.3)	11 (0.8)	0.041
**Waterpipe**				
No	2 835 (97.9)	1 533 (97.9)	1 302 (97.8)	0.997
Occasional	60 (2.1)	32 (2.0)	28 (2.1)	0.895
Daily	2 (0.1)	1 (0.1)	1 (0.1)	0.910
**Number of tobacco types used**				
1	830 (79.2)	406 (85.7)	424 (73.9)	<0.0001
>1	218 (20.8)	69 (14.3)	150 (26.1)	
**Secondhand smoke exposure**				
Yes[1]	89 (3.1)	49 (3.2)	40 (3.1)	0.852
		Mean (range)		p-value
Age when starting to smoke cigarettes regularly	17.1 (12-22)	16.9 (12-22)	17.4 (12-22)	0.013[b]
Mean nr cigarettes/week (occasional smokers)	8.3 (1-150)	7.7 (0-50)	9.0 (0-150)	0.856[c]
Mean nr cigarettes/day (daily smokers)	9.0 (1-30)	8.6 (1-25)	9.7 (1-30)	0.085[c]
Age when starting to snus regularly	18.5 (13-24)	20.2 (15-23)	18.0 (13-24)	<0.0001[b]
Mean nr boxes snus/month (occasional snus users)	2.5 (0-15)	1.8 (0-10)	3.0 (0-15)	0.003[c]
Mean nr boxes snus/week (daily snus users)	3.6 (0.5-10)	3.0 (0.5-7)	3.7 (0.5-10)	0.004[c]

[a]: P-value from Pearson χ^2 test.
[b]: P-value from two-tailed t-test.
[c]: P-value from Wilcoxon rank-sum test
[1]: Defined as daily exposure to indoor tobacco smoke

For peer review only

For peer review only - http://bmjopen.bmj.com/site/about/guidelines.xhtml

Description of validation population

The sociodemographic characteristics of the validation population did not differ from the total study population (**Table 1**), apart from a slightly higher prevalence of men (50.0% vs. 47.0%) and current asthma (13.7% vs 11.3%). As a consequence of the inclusion criteria, self-reported tobacco use in the validation population was higher than in the total study population, with 51.0% using any tobacco (daily or occasionally). A summary of tobacco use in the validation population is displayed in **Table S1** in the supplement.

Urine cotinine levels

Cotinine levels by tobacco type is shown in **Figure 1** and detailed urine cotinine levels are presented in **Table S2** in the supplement. In total, 76.2% of the samples had cotinine levels above the LOD and 44.4% had levels over the 50 ng/ml cut-off for validation. For all types of tobacco, daily users had significantly higher median cotinine levels than occasional users; 2 094 ng/ml vs. 422 ng/ml ($p<0.0001$) for smokers, 3 599 ng/ml vs. 345 ng/ml ($p<0.0001$) for snus users and 2 749 ng/ml vs. 527 ng/ml ($p=0.019$) for e-cigarette users. This pattern remained after exclusion of participants with mixed tobacco use, although cotinine levels were generally lower in each group. Among mixed tobacco users the median cotinine level was 3 073 ng/ml. Significantly higher cotinine levels were observed among high compared to low consumption of daily cigarette smoking and snus use (≥10 cigarettes/day or ≥ 4 boxes/week) after excluding other tobacco use (**Figures S2 and S3**). Men had higher cotinine levels than women among occasional cigarette smokers (1 595 ng/ml vs. 159 ng/ml, $p=0.0003$) and daily snus users (3 900 ng/ml vs. 2 762 ng/ml, $p=0.006$). However, no significant gender differences were observed after excluding mixed tobacco users. The median level for non-users was 1.6 ng/ml. Non-tobacco users exposed to daily secondhand smoke (n=7) had a median cotinine level of 2.47 ng/ml.

Validation of self-reported tobacco use

Table 3 displays validation statistics of self-reported tobacco use against urinary cotinine levels, by category of tobacco use. The overall agreement was 86.5%, the overall false negative rate (i.e., underreporting) was 3.5% and the overall false positive rate (i.e., overreporting) was 10.0%. Among

For peer review only - http://bmjopen.bmj.com/site/about/guidelines.xhtml

the underreporters, 40.0% reported former tobacco use and among the overreporters, 88.0% reported occasional tobacco use.

For both daily cigarette smoking and snus use, the agreement between self-reports and urinary cotinine was classified according to McHugh as strong (κ=0.824 and κ=0.861, respectively), with high sensitivity (80.2% and 83.6%, respectively) and specificity (98.3% and 99.1%, respectively). For occasional smoking and snus use, agreement and sensitivity were lower at the chosen cut-off level (κ=0.618 and κ=0.573, respectively), while specificity remained high. For any use of cigarettes and snus (daily and occasional combined) the agreement, sensitivity and specificity were high, with moderate agreement for smoking (κ=0.762) and strong for snus (κ=0.814). For any e-cigarette use, the agreement was weak (κ=0.576), with lower sensitivity (60.7%) compared to any smoking (86.6%) and any snus use (86.8%), but with high specificity (94.6%). No separate analyses were made for daily or occasional use for e-cigarettes, due to few daily users. However, all daily users of e-cigarettes had cotinine levels above the 50 ng/ml cut-off. NPV was consistently high across tobacco categories, while PPV was high for daily tobacco use (94.7% for cigarettes and 97.8% for snus) and somewhat lower for occasional use (68.0% for cigarettes and snus) and any e-cigarette use (67.5%).

Stratified analyses by gender revealed higher validity among men for snus and e-cigarette use, while no major gender differences were observed for cigarette smoking (see supplement **Table S3** for details).

Sensitivity analyses

When excluding mixed tobacco users, agreement was generally somewhat lower compared to the analyses with the full validation population (see supplement **Table S4** for details). In contrast, the agreement tended to be somewhat higher after exclusion of participants who provided urine samples ≥6 weeks after answering the questionnaire, however the interpretation of Kappa values was generally not influenced (see **Table S5** for details).

For peer review only

Table 3. Self-reported tobacco use against urinary cotinine levels by tobacco use.

Self-reports	Urinary cotinine level						
**Cigarette smoking**					**Daily**	**Occasional**	**Any**
	<50 ng/ml	≥50 ng/ml	*Total*	κ:	0.824	0.618	0.762
No	455	35	*490*	Sensitivity:	80.2%	70.8%	86.6%
Occasional	40	85	*125*	Specificity:	98.3%	91.9%	90.5%
Daily	8	142	*150*	PPV:	94.7%	68.0%	82.5%
Total	*503*	*262*	*765*	NPV:	92.9%	92.9%	92.9%
**Snus use**					**Daily**	**Occasional**	**Any**
	<50 ng/ml	≥50 ng/ml	*Total*	κ:	0.861	0.573	0.814
No	455	35	*490*	Sensitivity:	83.6%	59.3%	86.8%
Occasional	24	51	*75*	Specificity:	99.1%	95.0%	94.2%
Daily	4	178	*182*	PPV:	97.8%	68.0%	89.1%
Total	*483*	*264*	*747*	NPV:	92.9%	92.9%	92.9%
**E-cigarette use**					**Any**		
	<50 ng/ml	≥50 ng/ml	*Total*	κ:	0.576		
No	455	35	*490*	Sensitivity:	60.7%		
Occasional	26	45	*71*	Specificity:	94.6%		
Daily	0	9	*9*	PPV:	67.5%		
Total	*481*	*89*	*570*	NPV:	92.9%		

Cigarette smoking: Analysis including all self-reported cigarette smokers and all non-tobacco users.
Snus use: Analysis including all self-reported snus users and all non-tobacco users.
E-cigarette use: Analysis including all self-reported e-cigarette users and all non-tobacco users.
Daily: Results from analyses excluding occasional tobacco users.
Occasional: Results from analyses excluding daily tobacco users.
Any: Results from analyses combining daily and occasional tobacco users.
κ: Cohen's kappa coefficient.
PPV: Positive predictive value
NPV: Negative predictive value

Prediction model for underreporting

Using the backward stepwise selection approach, the factors former smoking, former snus use and time between questionnaire and urine sampling were included in the final prediction model. Former snus users were more likely to underreport tobacco use (odds ratio (OR): 5.7, 95% CI: 2.2, 15,0), and a non-significant association was observed for former smoking (OR: 2.1, 95% CI: 0.9, 4.6). Providing the urine sample more than 6 weeks after the questionnaire, as compared to within 6 weeks after the questionnaire, was also predictive of underreporting (OR: 1.5, 95% CI: 0.7, 2.9), although not

For peer review only - http://bmjopen.bmj.com/site/about/guidelines.xhtml

statistically significant. The performance of the prediction model, estimated by area under ROC curve (AUC), was 0.66.

DISCUSSION

Among young adults from a Swedish population-based cohort study, we observed high validity of self-reported tobacco use, particularly among daily users, while somewhat lower for occasional use as well as for e-cigarette use at the cut-off level of 50 ng/ml urinary cotinine. Specificity and NPV of the questionnaire were consistently high across tobacco type and frequency of use, while sensitivity and PPV varied and was somewhat lower, especially for occasional tobacco use. Former use of tobacco and a longer time between answering the questionnaire and providing a urine sample were predictive of underreporting, although former snus use was the only statistically significant prediction factor. Further, there were significant gender differences in tobacco use. Daily consumption of snus was high among men, while daily cigarette smoking was more common among women. Daily e-cigarette use was uncommon among both sexes. Daily snus users had the highest urine cotinine levels.

This study has several strengths, including a large and well-characterized study population from a population-based cohort, which provided power for the statistical analyses and allowed for stratification by tobacco type and gender. Furthermore, the study had repeatedly collected information on tobacco exposure, as well as sociodemographic factors. However, a limitation of the study design is that the questionnaire and the urine sampling were not conducted at the same occasion, and some participants may have changed their tobacco habits between the two assessments. The importance of time between the two assessments was apparent from both the sensitivity analysis as well as from the prediction model. Another limitation is the lack of information specific tobacco brands or types, such as nicotine-free products, which may further cause misclassification of certain self-reported tobacco users. Similarly, use of nicotine replacement products may explain cotinine levels above the cut-off level among some of the former tobacco users(17). However, only one participant in the validation populations reported using nicotine replacement products at the day of urine sampling and was thus excluded from the analyses. Further, selection bias cannot be entirely ruled out as the validation

For peer review only

For peer review only - http://bmjopen.bmj.com/site/about/guidelines.xhtml

population was selected from those attending the clinical examination, if non-attendance at clinical examination was related to accuracy in self-reporting.

Our study shows similar prevalence of tobacco use among young adults compared to data for the age group 16-29 years from Swedish national surveys conducted in 2016, 2018 and 2020(18,19). From 2016-2020, daily smoking was reported by 4-8% of young adults, daily snus use by 10-11% and any e-cigarette use at 2-3%, as compared to 7%, 11%, and 4%, respectively, in our study. On the other hand, occasional smoking was somewhat higher in our study (13% vs. 8-11%). Further, the observed gender differences in our study were similar to those of a recent study among Swedish 19-yearolds(20) and with figures from the Swedish national survey, describing higher snus and e-cigarette use among young men compared to women, but no difference in smoking prevalence between the genders(18,19). However, a recent study on pooled data from two population based Swedish cohort also found that cigarette smoking was more common among women across the age group 20-75 years(7).

Urinary cotinine levels were consistent with self-reported frequency of tobacco use. Daily users had higher cotinine levels than occasional users, and a dose-response trend was observed for the amount of cigarettes and snus consumed. Daily snus users had the highest median cotinine levels, followed by e-cigarette and cigarette users. Smokeless tobacco users have previously been found to have higher urinary cotinine levels than cigarettes smokers(21,22), while the difference between cigarette smokers and e-cigarette users appear less consistent(22–24). However, e-cigarette users were few in our study and the resulting cotinine levels may not be generalizable to other populations.

The high validity of self-reported tobacco use found in our study is consistent with previous findings. Several validation studies on self-reported cigarette smoking have found high correlation between urinary cotinine and self-reported smoking status in population-based cohorts, including young adult populations(25–30). In a cohort of young men and women in the United States (aged 18-30 years), the overall agreement between self-reported cigarette smoking (at least 5 cigarettes per week) and urinary cotinine at a cut-off level of 14 ng/ml was 95.8%(30). Similarly, high sensitivity (93%) and specificity (99%) for self-reported smoking compared to a cotinine cut-off level of 25 ng/ml was found in a cohort of Malaysian male university students(29).

For peer review only - http://bmjopen.bmj.com/site/about/guidelines.xhtml

In a previous validation study of self-reported snus use, high validity was found among Swedish pregnant women and adolescents (mean age 15 years)(6,31).

To date, few studies have validated self-reported e-cigarette use against biomarkers in population-based cohorts. Boykan et al.(32) found a low (37%) concordance among adolescent self-reported e-cigarette users in the past week. The lower agreement observed for self-reported e-cigarette use in our study as compared to cigarettes and snus is likely a consequence of that most of the e-cigarette use was occasional, which was also true in the study by Boykan et al. In fact, in our study all daily e-cigarette users had cotinine levels above the cut-off value, although they were very few. Further studies with larger sample sizes are needed to assess validity of self-reported e-cigarette use more confidently.

Some discrepancies between genders were observed for validity of self-reporting where men had generally higher validity, particularly for snus and e-cigarette use where the prevalence was higher among men. However, gender was not related to underreporting. In studies conducted in the United Arab Emirates, Georgia and South Korea, much lower validity for self-reported smoking was found among women compared to men, however smoking rates were also much lower among women it these studied(25,33,34). In contrast, in studies from Canada, the United States and Mexico, where smoking rates were more even between genders, similar validity was also observed(28,30,35). These discrepancies may be related to cultural differences between the geographic regions of the studies.

We found low rates of underreporting in our study population, in a similar or lower range than other study populations from the general population(25–28,30,31,36). However, it should be noted that rates of underreporting tend to be notably higher among certain patient groups, for example chronic obstructive pulmonary disease patients(37), head and neck cancer patients(38,39), cardiovascular disease patients(40,41), as well as among pregnant women(6,42,43), especially when investigating smoking cessation. Although some nicotine exposure may occur from secondhand smoke exposure or diet(44,45), such exposure is unlikely to cause misclassification at a cut-off level 50 ng/ml. Instead, the strongest predictive factor of underreporting was former tobacco use. This is in line with previous findings(28,30), and may be explained by relapse or use of other nicotine products during the study period. Some overreporting was also found in the validation population, particularly among occasional

For peer review only

For peer review only - http://bmjopen.bmj.com/site/about/guidelines.xhtml

tobacco users. Lower agreement in this group is expected compared to daily tobacco users at the same biochemical cut-off level, as their nicotine exposure would vary more over time. The same discrepancies between these groups and similar level of agreement between self-reported occasional cigarette smoking and serum cotinine was observed in a recent NHANES study(36). As the half-life of cotinine is 12-20 hours(2), it is likely that occasional users will have urinary cotinine levels similar to non-users only a few days after their latest use. Validity among occasional tobacco users is therefore likely not fully captured with a single urine sampling or at the chosen cut-off level.

As the validation was performed in a subsample of the BAMSE cohort, the results may not be entirely representative of the full cohort. Despite being enriched with tobacco users, the validation population was generally representative of the study population in sociodemographic factors, which indicates high validity in the whole cohort. However, as there were proportionally more non-tobacco users in the study population than the validation population, the rate of underreporting may be somewhat higher in the study population. Similarly, the number of overreporters in the study population may be lower, due to tobacco users being proportionally fewer. Still, the results of the validation analyses will be applicable to future research conducted on tobacco exposure in the BAMSE cohort. From a broader perspective, these results together with previous findings indicate that self-reported data on tobacco consumption can be used to accurately assess exposure for young adults. Questionnaires are important tools to study health effects of tobacco and can be used to meet the demand on more research on both e-cigarettes and snus. Although snus is banned in most EU countries(46) and is not a globally used type of tobacco, there has been a rise in snus sales in the USA over the past years(47). This spread on the market increases the need to uncover adverse health effects of snus, in order to promote global public health.

CONCLUSION

In this study, we observed significant gender differences in tobacco use among Swedish young adults, with a notably high prevalence of daily snus consumption among young men. Snus users had the highest levels of urinary cotinine. Further, we found high validity of self-reported tobacco use,

For peer review only - http://bmjopen.bmj.com/site/about/guidelines.xhtml

For peer review only

particularly among daily tobacco users, with very low rates of underreporting. These results indicate that self-reported tobacco use is valid for exposure assessment among young adults, which is important for future research on health effects of tobacco and nicotine in this age group.

List of abbreviations

E-cigarettes: Electronic cigarettes

LC-MS/MS: Liquid chromatography tandem mass spectrometry

BMI: Body mass index

LOD: Limit of detection

SG: Specific gravity

PPV: Positive predictive value

NPV: Negative predictive value

OR: Odds ratio

AUC: Area under ROC curve

Caption Figure 1. A: Density adjusted urinary cotinine levels by self-reported tobacco use. No tobacco (n=490); Cigarettes, daily (n=150); Cigarettes, occasional (n=125); Snus, daily (n=182); Snus, occasional (n=75), E-cigarettes, daily (n=9); E-cigarettes, occasional (n=71); Waterpipe, any (n=26). Including participants with mixed tobacco use.

B: Density adjusted urinary cotinine levels among participants without mixed tobacco use as well as all mixed users. Cigarettes, daily (n=112); Cigarettes, occasional (n=64); Snus, daily (n=119); Snus, occasional (n=55), E-cigarettes, daily (n=7); E-cigarettes, occasional (n=25); Waterpipe, any (n=9); Mixed tobacco (155).

For peer review only

Acknowledgement

We thank the children and parents participating in the BAMSE cohort and all staff involved in the study through the years. Moreover, we wish to thank Anna Rönnholm, Marie Bengtsson, and Åsa Amilon at the Division of Occupational and Environmental Medicine at Lund University, Sweden, for performing the analyses of chemicals.

Funding

This work was supported by the Swedish Research Council (VR, grant number 2018-02524); the Swedish Heart and Lung Foundation; the Swedish Research Council for Sustainable Development (FORMAS, grant number 2016-01646); the Swedish Research Council for Working Life and Social Welfare; the Swedish Asthma and Allergy Association Research Foundation; the Swedish Environmental Protection Agency (grant numbers NV-09284-13 and NV-00175-15); Region Stockholm (ALF project, and for cohort and database maintenance); the European Research Council (grant number 757919); and Karolinska Institutet.

Competing interests

The authors declare that they have no competing interests.

Author's contribution

AZ took part in the study design, performed the formal analyses and interpretation of the results, created tables and figures, and wrote the original draft. SS contributed to revising the manuscript. LP contributed to revising the manuscript and provided funding. PL contributed to revising the manuscript and provided funding. GP contributed to revising the manuscript. NA provided data curation, assistance with the formal analysis and contributed to revising the manuscript. CL provided resources for and was responsible for the urine analysis and contributed to revising the manuscript. AG contributed to revising the manuscript. IK contributed to revising the manuscript. EM contributed to revising the manuscript and provided funding. SE took part in the study design, provided data curation and assistance with the formal analysis, contributed to the supervision of the project was a major contributor in revising the manuscript. AB had a major role in study design, methodology, project

For peer review only - http://bmjopen.bmj.com/site/about/guidelines.xhtml

administration and supervision of the project, provided funding and resources and was a major contributor in revising the manuscript. All authors read and approved the final manuscript.

Availability of data and materials

The datasets generated and/or analyzed during the current study are not publicly available, due to the dataset containing sensitive personal data, but are available from the corresponding author on reasonable request.

Ethical approval statement

The study was approved by the Regional Ethics Committee at Karolinska Institutet in Stockholm, Sweden (Dnr 2016/1380-31/2).

References

1. Gorber SC, Schofield-Hurwitz S, Hardt J, Levasseur G, Tremblay M. The accuracy of self-reported smoking: A systematic review of the relationship between self-reported and cotinine-assessed smoking status. Nicotine Tob Res. 2009;11(1):12–24.
2. Kim S. Overview of cotinine cutoff values for smoking status classification. Int J Environ Res Public Health. 2016;13(12).
3. Agaku IT, King BA. Validation of Self-Reported Smokeless Tobacco Use by Measurement of Serum Cotinine Concentration Among US Adults. Am J Epidemiol. 2014;180(7):749–54.
4. Swedish Public Health Agency. Vuxnas bruk av tobaks- och nikotinprodukter [Internet]. 2022 [cited 2023 Jan 25]. Available from: https://www.folkhalsomyndigheten.se/livsvillkor-levnadsvanor/andts/utveckling-inom-andts-anvandning-och-ohalsa/anvandning/anvandning-av-tobaks-och-nikotinprodukter/vuxnas-bruk-av-tobaks--och-nikotinprodukter/
5. Siddiqi K, Husain S, Vidyasagaran A, Readshaw A, Mishu MP, Sheikh A. Global burden of disease due to smokeless tobacco consumption in adults: An updated analysis of data from 127

For peer review only - http://bmjopen.bmj.com/site/about/guidelines.xhtml

For peer review only

countries. BMC Med. 2020;18(1).

6. Gunnerbeck A, Raaschou P, Cnattingius S, Edstedt Bonamy AK, Wickström R. Maternal snuff use and cotinine in late pregnancy—A validation study. Acta Obstet Gynecol Scand. 2018;97(11):1373–80.

7. Hedman L, Backman H, Stridsman C, Bosson JA, Lundbäck M, Lindberg A, et al. Association of Electronic Cigarette Use With Smoking Habits, Demographic Factors, and Respiratory Symptoms. JAMA Netw open. 2018;1(3):e180789.

8. Rom O, Pecorelli A, Valacchi G, Reznick AZ. Are E-cigarettes a safe and good alternative to cigarette smoking? Ann N Y Acad Sci. 2015;1340(1):65–74.

9. Gotts JE, Jordt SE, McConnell R, Tarran R. What are the respiratory effects of e-cigarettes? BMJ. 2019;366:1–31.

10. Melén E, Bergström A, Kull I, Almqvist C, Andersson N, Asarnoj A, et al. Male sex is strongly associated with IgE-sensitization to airborne but not food allergens: Results up to age 24 years from the BAMSE birth cohort. Clin Transl Allergy [Internet]. 2020;10(1):1–7. Available from: https://doi.org/10.1186/s13601-020-00319-w

11. Wickman M, Kull I, Pershagen G, Sl N, Bamse T. The BAMSE Project : presentation of a prospective longitudinal birth cohort study. Pediatr Allergy Immunol. 2002;13:11–3.

12. Axelsson J, Rylander L, Rignell-Hydbom A, Lindh CH, Jönsson BAG, Giwercman A. Prenatal phthalate exposure and reproductive function in young men. Environ Res [Internet]. 2015;138:264–70. Available from: http://dx.doi.org/10.1016/j.envres.2015.02.024

13. Hauser R, Meeker JD, Park S, Silva MJ, Calafat AM. Temporal variability of urinary phthalate metabolite levels in men of reproductive age. Environ Health Perspect. 2004;112(17):1734–40.

14. Benowitz NL, Jacob P, Ahijevych K, Jarvis MJ, Hall S, LeHouezec J, et al. SRNT Subcommittee on Biochemical Verification. Biochemical verification of tobacco use and cessation. Nicotine Tob Res. 2002;4(2):149–59.

For peer review only - http://bmjopen.bmj.com/site/about/guidelines.xhtml

15. McHugh ML. Interrater reliability: the kappa statistics. Biochem Medica. 2012;22(3):276–82.

16. Kirkwood BR, Sterne JAC. Essential Medical Statistics. 2nd ed. Malden, Massachusetts: Blackwell Publishing Company; 2003. 341 p.

17. Hickson C, Lewis S, Campbell KA, Cooper S, Berlin I, Claire R, et al. Comparison of nicotine exposure during pregnancy when smoking and abstinent with nicotine replacement therapy: systematic review and meta-analysis. Addiction. 2019;114(3):406–24.

18. Swedish Public Health Agency. Vuxnas bruk av cigaretter, snus och e-cigaretter [Internet]. 2020 [cited 2020 Nov 13]. Available from: https://www.folkhalsomyndigheten.se/livsvillkor-levnadsvanor/andts/utveckling-inom-andts-anvandning-och-ohalsa/bruk/tobak-och-liknande-produkter/vuxnas-bruk-av-cigaretter-snus-och-e-cigaretter/

19. Swedish Public Health Agency. Tobakskonsumtion (självrapporterat) efter ålder, kön och år. Andel (procent). [Internet]. 2020 [cited 2021 May 3]. Available from: http://fohm-app.folkhalsomyndigheten.se/Folkhalsodata/pxweb/en/B_HLV/B_HLV__aLevvanor__aagLevvanortobak/hlv1tobaald.px/

20. Hedman L, Backman H, Stridsman C, Lundbäck M, Andersson M, Rönmark E. Predictors of electronic cigarette use among Swedish teenagers: A population-based cohort study. BMJ Open. 2020;10(12).

21. Çok I, Öztürk R. Urinary cotinine levels of smokeless tobacco (Maraş powder) users1. Hum Exp Toxicol. 2000;19(11):650–5.

22. Lorkiewicz P, Riggs DW, Keith RJ, Conklin DJ, Xie Z, Sutaria S, et al. Comparison of Urinary Biomarkers of Exposure in Humans Using Electronic Cigarettes, Combustible Cigarettes, and Smokeless Tobacco. Nicotine Tob Res. 2019;21(9):1228–38.

23. Göney G, Çok İ, Tamer U, Burgaz S, Şengezer T. Urinary cotinine levels of electronic cigarette (e-cigarette) users. Toxicol Mech Methods. 2016;26(6):414–8.

24. Park MB, Choi JK. Differences between the effects of conventional cigarettes, e-cigarettes and

For peer review only

For peer review only - http://bmjopen.bmj.com/site/about/guidelines.xhtml

dual product use on urine cotinine levels. Tob Induc Dis. 2019;17(February):1–9.

25. Al-Houqani M, Leinberger-Jabari A, Al naeemi A, Al Junaibi A, Al Zaabi E, Oumeziane N, et al. Patterns of tobacco use in the United Arab Emirates Healthy Future (UAEHFS) pilot study. PLoS One. 2018;13(5).

26. Hoseini M, Yunesian M, Nabizadeh R, Yaghmaeian K, Parmy S, Gharibi H, et al. Biomonitoring of tobacco smoke exposure and self-reported smoking status among general population of Tehran, Iran. Environ Sci Pollut Res [Internet]. 2016;23(24):25065–73. Available from: http://dx.doi.org/10.1007/s11356-016-7619-8

27. Pearce MS, Mann KD, Singh G, Davison B, Sayers SM. Prevalence and validity of self-reported smoking in Indigenous and non-Indigenous young adults in the Australian Northern Territory. BMC Public Health. 2014;14(1):1–7.

28. Wong SL, Shields M, Leatherdale S, Malaison E, Hammond D, Wong SL, et al. Assessment of validity of self-reported smoking status. Heal Reports. 2012;23(1).

29. Man CN, Fathelrahman AI, Harn GL, Lajis R, Samin ASM, Omar M, et al. Correlation between urinary nicotine, cotinine and self-reported smoking status among educated young adults. Environ Toxicol Pharmacol. 2009;28(1):92–6.

30. Wagenknecht LE, Burke GL, Perkins LL, Haley NJ, Friedman GD. Misclassification of smoking status in the CARDIA study: A comparison of self-report with serum cotinine levels. Am J Public Health. 1992;82(1):33–6.

31. Post A, Gilljam H, Rosendahl I, Meurling L, Bremberg S, Galanti MR. Validity of self reports in a cohort of Swedish adolescent smokers and smokeless tobacco (snus) users. Tob Control. 2005;14(2):114–7.

32. Boykan R, Messina CR, Chateau G, Eliscu A, Tolentino J, Goniewicz ML. Self-Reported Use of Tobacco, E-cigarettes, and Marijuana Versus Urinary Biomarkers. Pediatrics [Internet]. 2019 [cited 2021 Oct 26];143(5):e20183531. Available from:

For peer review only

For peer review only - http://bmjopen.bmj.com/site/about/guidelines.xhtml

https://doi.org/10.1542/peds.2018-3531

33. Williams J, Rakovac I, Loyola E, Sturua L, Maglakelidze N, Gamkrelidze A, et al. A comparison of self-reported to cotinine-detected smoking status among adults in Georgia. Eur J Public Health. 2020;30(5):1007–12.

34. Hwang JH, Kim JY, Lee DH, Jung HG, Park SW. Underestimation of self-reported smoking prevalence in Korean adolescents: Evidence from gold standard by combined method. Int J Environ Res Public Health. 2018;15(4):1–10.

35. Valladolid-López MDC, Barrientos-Gutiérrez T, Reynales-Shigematsu LM, Thrasher JF, Peláez-Ballestas I, Lazcano-Ponce E, et al. Evaluating the validity of self-reported smoking in Mexican adolescents. BMJ Open. 2015;5(10):1–8.

36. Guitérrez-Torres DS, Wang L, Blount BC, Xia B, Sosnoff CS, Shiels MS, et al. Concentrations of Cotinine and 4-(Methylnitrosamino)-1-(3-Pyridyl)-1-Butanol (NNAL) in U.S. Non-Daily Cigarette Smokers. Cancer Epidemiol Biomarkers Prev. 2021;30:1165–74.

37. Hilberink SR, Jacobs JE, van Opstal S, van der Weijden T, Keegstra J, Kempers PLJ, et al. Validation of smoking cessation self-reported by patients with chronic obstructive pulmonary disease. Int J Gen Med. 2011;4:85–90.

38. Alberg AJ, Worley ML, Tooze JA, Hatcher JL, Carpenter MJ, Day TA, et al. The validity of self-reported recent smoking in head and neck cancer surgical patients. Otolaryngol - Head Neck Surg (United States). 2015;153(6):990–5.

39. Gritz ER, Carr CR, Rapkin D, Abemayor E, Chang LJC, Wong WK, et al. Predictors of Long-Term Smoking Cessation in Head and Neck Cancer Patients. Cancer Epidemiol Biomarkers Prev. 1993;2(3):261–70.

40. Ebner N, Földes G, Szabo T, Tacke M, Fülster S, Sandek A, et al. Assessment of serum cotinine in patients with chronic heart failure: Self-reported versus objective smoking behaviour. Clin Res Cardiol. 2013;102(2):95–101.

For peer review only

For peer review only - http://bmjopen.bmj.com/site/about/guidelines.xhtml

41. Wilson DK, Wallston KA, King JE, Smith MS, Heim C. Validation of smoking abstinence in newly diagnosed cardiovascular patients. Addict Behav. 1993;18(4):421–9.

42. England LJ, Grauman A, Qian C, Wilkins DG, Schisterman EF, Yu KF, et al. Misclassification of maternal smoking status and its effects on an epidemiologic study of pregnancy outcomes. Nicotine Tob Res. 2007;9(10):1005–13.

43. Ashford K, Wiggins A, Rayens E, Assef S, Fallin A, Rayens MK. Perinatal biochemical confirmation of smoking status by trimester. Nicotine Tob Res. 2017;19(5):631–5.

44. Haufroid V, Lison D. Urinary cotinine as a tobacco-smoke exposure index. A minireview. Int Arch Occup Environ Health. 1998;71(3):162–8.

45. Davis RA, Stiles MF, deBethizy JD, Reynolds JH. Dietary nicotine: A source of urinary cotinine. Food Chem Toxicol. 1991;29(12):821–7.

46. European Commission. Revision of the Tobacco Products Directive [Internet]. Vol. 2014. 2014. Available from: https://ec.europa.eu/health//sites/health/files/tobacco/docs/dir_201440_en.pdf%0Ahttps://ec.europa.eu/health/sites/health/files/tobacco/docs/dir_201440_en.pdf%0Ahttps://ec.europa.eu/health//sites/health/files/tobacco/docs/dir_201440_en.pdf%0Ahttp://ec.eur

47. Federal Trade Commission. Federal Trade Commission Smokeless Tobacco Report for 2020 [Internet]. 2021. Available from: https://www.ftc.gov/system/files/documents/reports/federal-trade-commission-cigarette-report-2020-smokeless-tobacco-report-2020/p114508fy20smokelesstobacco.pdf

For peer review only

For peer review only - http://bmjopen.bmj.com/site/about/guidelines.xhtml

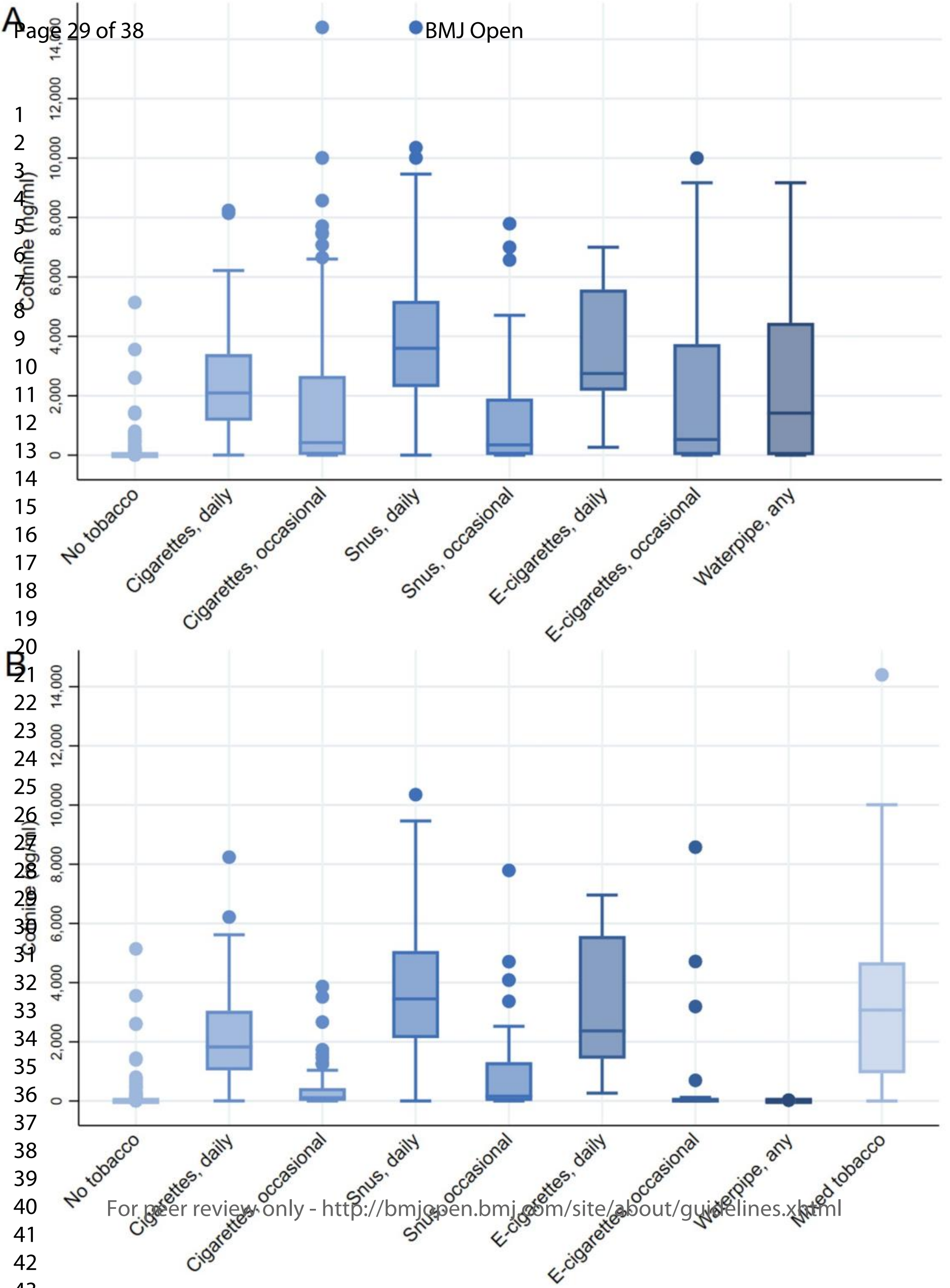
A
Cotinine (ng/ml)
0
2,000
4,000
6,000
8,000
10,000
12,000
14,000
No tobacco
Cigarettes, daily
Cigarettes, occasional
Snus, daily
Snus, occasional
E-cigarettes, daily
E-cigarettes, occasional
Waterpipe, any
B
Cotinine (ng/ml)
0
2,000
4,000
6,000
8,000
10,000
12,000
14,000
No tobacco
Cigarettes, daily
Cigarettes, occasional
Snus, daily
Snus, occasional
E-cigarettes, daily
E-cigarettes, occasional
Waterpipe, any
Mixed tobacco

Assessing tobacco use in Swedish young adults from self-report and urinary cotinine: a validation study using the BAMSE birth cohort

Supplementary information

Anna Zettergren, Shanzina Iasmin Sompa, Lena Palmberg, Petter Ljungman, Göran Pershagen, Niklas Andersson, Christian H Lindh, Antonios Georgelis, Inger Kull, Erik Melén, Sandra Ekström, Anna Bergström

For peer review only

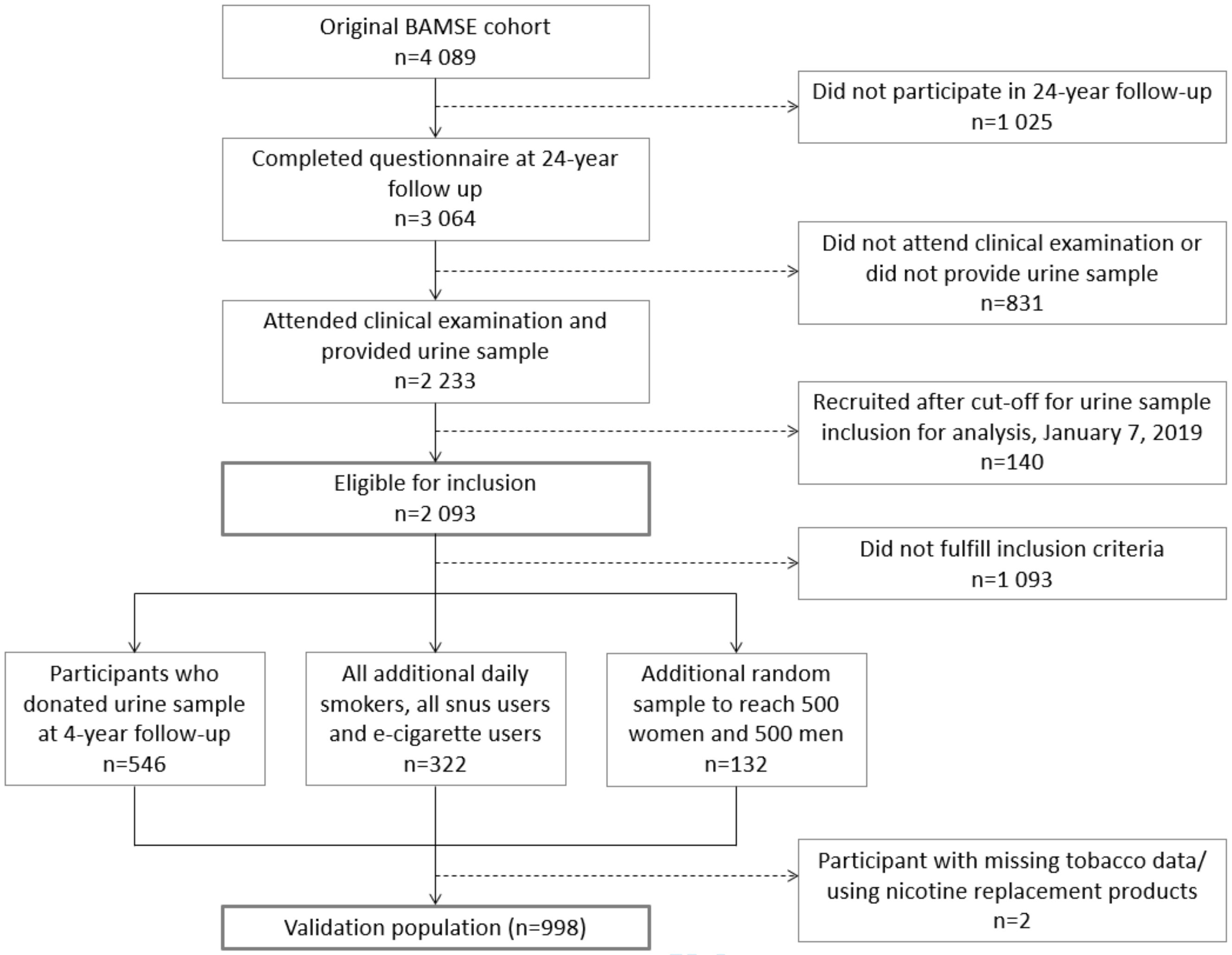

Figure S1. Overview of the selection process of the validation population for urine sample analysis of cotinine.

Variable definitions:

Age: Age in years at questionnaire, as calculated from date of birth.

Parental socioeconomic status: Household economic status at enrollment in the BAMSE study. Categorized as i) blue collar worker or ii) white collar worker, based on the highest employment category of the parents.

Any parent smoking at baseline: Any parent smoking at baseline of the BAMSE cohort. Based on self-reported questionnaire data from parents.

Education level: Participants highest completed education level at the time of the 24-year follow-up questionnaire. Categorized as i) elementary school or high school and ii) university or higher.

Occupation: Based on questionnaire data from 24-year follow-up. Categorized as i) student, ii) working or iii) other.

Body mass index (BMI): Calculated as kg/m^2 from weight and height measurements from the 24-year follow-up clinical examination.

Secondhand smoke exposure: Based on self-reported exposure from the 24-year follow-up questionnaire. Categorized as i) "yes" if daily exposed to indoor tobacco smoke (at home, work or other places), otherwise as ii) "no".

Asthma: Defined based on information from the 24-year follow-up questionnaire as "yes" if participants had a doctor's diagnosis of Asthma together with either symptoms of breathing difficulties in the last 12 months OR taking asthma medication in the past 12 months.

For peer review only

Table S1. Self-reported tobacco use in the validation population (n=998).

	All n=998	Women n=499	Men n=499	p-value[a] (women vs. men)
		n (%)		
**Cigarettes**				
Never	588 (59.0)	279 (56.0)	309 (61.9)	0.054
Former	135 (13.5)	60 (12.0)	75 (15.0)	0.165
Occasional	125 (12.5)	64 (12.8)	61 (12.2)	0.774
Daily	150 (15.0)	96 (19.2)	54 (10.8)	<0.0001
<10 cigarettes/day	*82 (55.4)*	*57 (60.0)*	*25 (47.2)*	*0.132*
≥10 cigarettes/day	*66 (44.6)*	*38 (40.0)*	*28 (52.8)*	
**Snus**				
Never	704 (70.5)	429 (86.0)	275 (55.1)	<0.0001
Former	37 (3.7)	5 (1.0)	32 (6.4)	<0.0001
Occasional	75 (7.5)	28 (5.6)	47 (9.4)	0.023
Daily	182 (18.2)	37 (7.4)	145 (29.1)	<0.0001
<4 boxes of snus/week	*99 (54.4)*	*27 (73.0)*	*72 (49.7)*	*0.011*
≥4 boxes of snus/week	*83 (45.6)*	*10 (27.0)*	*73 (50.3)*	
**E-cigarettes**				
Never	918 (92.0)	472 (94.6)	446 (89.4)	0.002
Occasional	71 (7.1)	24 (4.8)	47 (9.4)	0.005
Daily	9 (0.9)	3 (0.6)	6 (1.2)	0.315
**Waterpipe**				
Never	970 (97.4)	488 (97.8)	482 (97.0)	0.421
Occasional	25 (2.5)	10 (2.0)	15 (3.0)	0.306
Daily	1 (0.1)	1 (0.2)	0 (0)	0.318
Number of tobacco types used				
1	393 (77.4)	190 (84.1)	203 (72.0)	0.001
>1	115 (22.6)	36 (15.9)	79 (28.0)	
Secondhand smoke exposure				
Yes[1]	39 (4.0)	24 (4.9)	15 (3.1)	0.141

[a]: P-value from Pearson χ^2 test.
[b]: P-value from two-tailed t-test.
[c]: P-value from Wilcoxon rank-sum test
[1]: Defined as daily exposure to indoor tobacco smoke.

For peer review only

Table S2. Urinary cotinine levels (ng/ml) by tobacco use categories, adjusted for specific gravity.

	n	Median	Mean	SD	95th percentile	Min-max
**Cigarettes**						
Any cigarette smoking	275	1 465	2 089	2 176	6 215	0.1–14 399
Any cigarette smoking, mixed users excluded	*176*	*1 180*	*1 494*	*1 545*	*4 528*	*0.1–8 238*
Daily cigarette smoking	150	2 094	2 390	1 670	5 264	3.6–8 238
Daily cigarette smoking, mixed users excluded	*112*	*1 826*	*2 101*	*1 547*	*4 957*	*3.6–8 238*
Occasional cigarette smoking	125	422	1 728	2 621	7 449	0.1–14 399
Occasional cigarette smoking, mixed users excluded	*64*	*109*	*433*	*787*	*1 733*	*0.1–3 868*
**Snus**						
Any snus use	257	2 939	3 159	2 510	7 790	0.3–14 399
Any snus use, mixed users excluded	*174*	*2 566*	*2 834*	*2 368*	*7 381*	*0.3–10 350*
Daily snus use	182	3 599	3 950	2 346	8 051	1.7–14 399
Daily snus use, mixed users excluded	*119*	*3 449*	*3 726*	*2 125*	*7 859*	*1.7–10 350*
Occasional snus use	75	345	1 239	1 752	4 707	0.3–7 790
Occasional snus use, mixed users excluded	*55*	*161*	*903*	*1 500*	*4 088*	*0.3–7 790*
**E-cigarettes**						
Any e-cigarette smoking	80	874	2 159	2 697	8 359	0.3–9 999
Any e-cigarette smoking, mixed users excluded	*32*	*10.7*	*1 219*	*2 260*	*6 958*	*0.5–8 574*
Daily e-cigarette smoking	9	2 749	3 643	2 431	6 998	265–6 998
Daily e-cigarette smoking, mixed users excluded	*7*	*2 368*	*3 074*	*2 357*	*6 958*	*265–6 958*
Occasional e-cigarette smoking	71	527	1 971	2 686	8 574	0.3–9 999
Occasional e-cigarette smoking, mixed users excluded	*25*	*5.7*	*700*	*1 981*	*4 714*	*0.5–8 574*
**Waterpipe**						
Any waterpipe use	26	1 415	2 400	2 698	6 602	0.3–9 169
Any waterpipe use, mixed users excluded	*9*	*2.0*	*7.2*	*10.1*	*28.9*	*0.7–28.9*

For peer review only

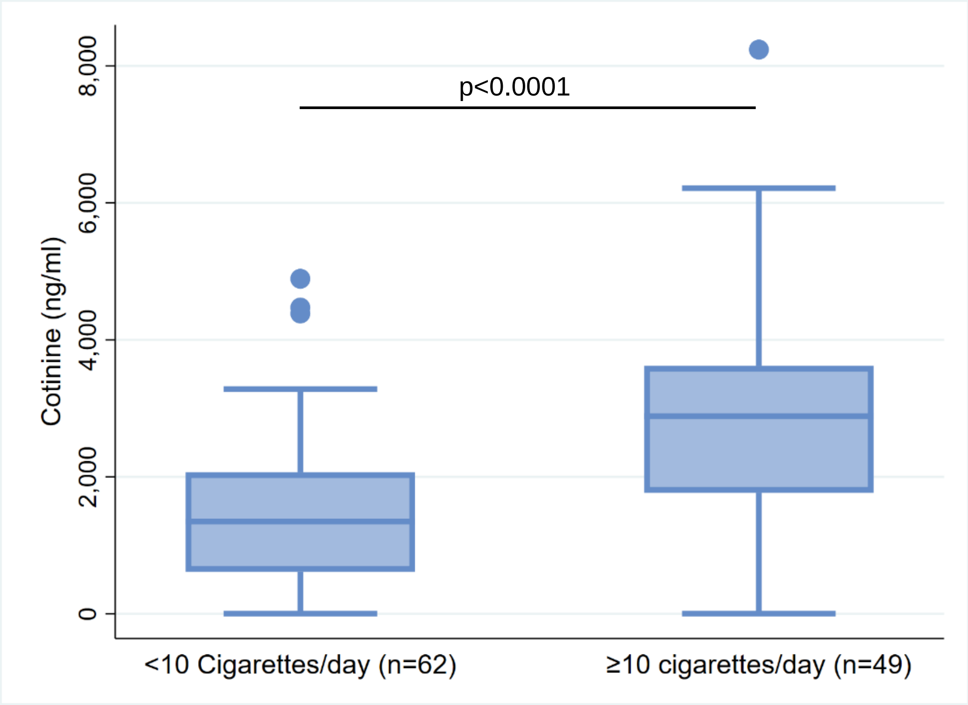

Figure S2. Urinary cotinine levels among daily cigarette smokers, by number of cigarettes per day. Participants with mixed tobacco use excluded.

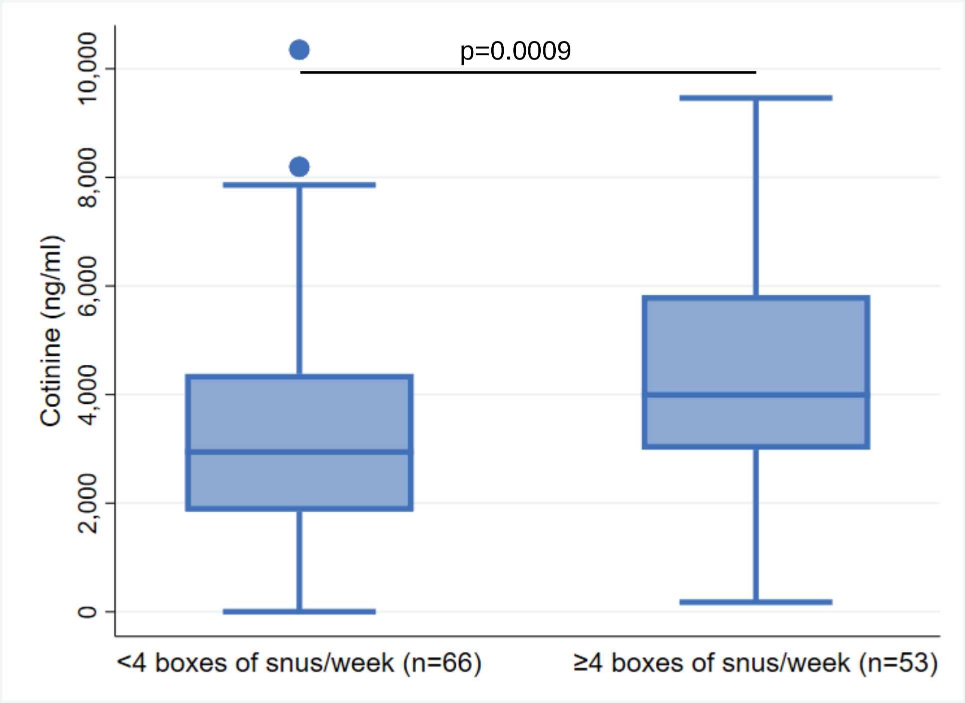

Figure S3. Urinary cotinine levels among daily snus users, by number of boxes snus per week. Participants with mixed tobacco use excluded.

Table S3. Self-reported tobacco use against urinary cotinine levels by type of tobacco and frequency of use, stratified by gender.

			Women	Men
Cigarettes	Daily	κ:	0.838	0.801
		Sensitivity:	83.3%	75.4%
		Specificity:	97.7%	99.0%
		PVV:	93.8%	96.3%
		NPV:	93.4%	92.2%
	Occasional	κ:	0.592	0.643
		Sensitivity:	69.5%	72.1%
		Specificity:	91.7%	92.2%
		PVV:	64.1%	72.1%
		NPV:	93.4%	92.2%
	Any	κ:	0.764	0.760
		Sensitivity:	87.9%	85.0%
		Specificity:	89.8%	91.3%
		PVV:	81.9%	83.5%
		NPV:	93.4%	92.2%
Snus	Daily	κ:	0.726	0.898
		Sensitivity:	65.4%	89.4%
		Specificity:	98.8%	99.5%
		PVV:	91.9%	99.3%
		NPV:	93.4%	92.2%
	Occasional	κ:	0.461	0.640
		Sensitivity:	47.1%	67.3%
		Specificity:	95.5%	94.3%
		PVV:	57.1%	74.5%
		NPV:	93.4%	92.2%
	Any	κ:	0.691	0.853
		Sensitivity:	73.5%	91.3%
		Specificity:	94.4%	93.9%
		PVV:	76.9%	93.2%
		NPV:	93.4%	92.2%
E-cigarette use	Any	κ:	0.418	0.658
		Sensitivity:	43.8%	70.2%
		Specificity:	95.1%	93.9%
		PVV:	51.9%	75.5%
		NPV:	93.4%	92.2%

Cigarette smoking: Analysis including all self-reported cigarette smokers and all non-tobacco users.
Snus use: Analysis including all self-reported snus users and all non-tobacco users.
E-cigarette use: Analysis including all self-reported E-cigarette users and all non-tobacco users.
Daily: Results from analyses excluding occasional tobacco users.
Occasional: Results from analyses excluding daily tobacco users.
Any: Results from analyses combining daily and occasional tobacco users.
κ: Cohen's kappa coefficient.
PPV: Positive predictive value
NPV: Negative predictive value

For peer review only

Table S4. Self-reported tobacco use against urinary cotinine levels, excluding participants with mixed tobacco use or with missing data on waterpipe use (n=117 excluded).

Self-reports	Urinary cotinine level						
**Cigarette smoking**					**Daily**	**Occasional**	**Any**
	<50 ng/ml	≥50 ng/ml	*Total*				
No	455	35	*490*	κ:	0.784	0.469	0.725
Occasional	28	36	*64*	Sensitivity:	74.8%	50.7%	80.0%
Daily	8	104	*112*	Specificity:	98.3%	94.2%	92.7%
Total	*491*	*175*	*666*	PPV:	92.9%	56.3%	79.5%
				NPV:	92.9%	92.9%	92.9%
**Snus use**					**Daily**	**Occasional**	**Any**
	<50 ng/ml	≥50 ng/ml	*Total*				
No	455	35	*490*	κ:	0.825	0.478	0.775
Occasional	22	33	*55*	Sensitivity:	77.0%	48.5%	81.1%
Daily	2	117	*119*	Specificity:	99.6%	95.4%	95.0%
Total	*479*	*185*	*664*	PPV:	98.3%	60.0%	86.2%
				NPV:	92.9%	92.9%	92.9%
**E-cigarette use**					**Any**		
	<50 ng/ml	≥50 ng/ml	*Total*				
No	455	35	*490*	κ:	0.293		
Occasional	18	7	*25*	Sensitivity:	28.6%		
Daily	0	7	*7*	Specificity:	96.2%		
Total	*473*	*49*	*522*	PPV:	43.8%		
				NPV:	92.9%		

Cigarette smoking: Analysis including self-reported cigarette smokers without other tobacco use and all non-tobacco users.
Snus use: Analysis including self-reported snus users without other tobacco use and all non-tobacco users.
E-cigarette use: Analysis including self-reported e-cigarette users without other tobacco use and all non-tobacco users.
Daily: Results from analyses excluding occasional tobacco users.
Occasional: Results from analyses excluding daily tobacco users.
Any: Results from analyses combining daily and occasional tobacco users.
κ: Cohen's kappa coefficient.
PPV: Positive predictive value
NPV: Negative predictive value

For peer review only

Table S5. Self-reported tobacco use against urinary cotinine levels for participants who provided urine samples within 6 weeks after answering the questionnaire (n=507 included).

Self-reports	Urinary cotinine level						
**Cigarette smoking**					**Daily**	**Occasional**	**Any**
	<50 ng/ml	≥50 ng/ml	*Total*				
No	216	15	*231*	κ:	0.867	0.639	0.785
Occasional	22	47	*69*	Sensitivity:	84.4%	75.8%	89.5%
Daily	2	81	*83*	Specificity:	99.1%	90.8%	90.0%
Total	*240*	*143*	*383*	PPV:	97.6%	68.1%	84.2%
				NPV:	93.5%	93.5%	93.5%
**Snus use**					**Daily**	**Occasional**	**Any**
	<50 ng/ml	≥50 ng/ml	*Total*				
No	216	15	*231*	κ:	0.890	0.643	0.861
Occasional	8	26	*34*	Sensitivity:	87.0%	63.4%	89.4%
Daily	1	101	*102*	Specificity:	99.5%	96.4%	96.0%
Total	*225*	*142*	*367*	PPV:	99.0%	76.5%	93.4%
				NPV:	93.5%	93.5%	93.5%
**E-cigarette use**					**Any**		
	<50 ng/ml	≥50 ng/ml	*Total*				
No	216	15	*231*	κ:	0.602		
Occasional	15	25	*40*	Sensitivity:	66.7%		
Daily	0	5	*5*	Specificity:	93.5%		
Total	*231*	*45*	*276*	PPV:	66.7%		
				NPV:	93.5%		

Cigarette smoking: Analysis including all self-reported cigarette smokers and all non-tobacco users.
Snus use: Analysis including all self-reported snus users and all non-tobacco users.
E-cigarette use: Analysis including all self-reported E-cigarette users and all non-tobacco users.
Daily: Results from analyses excluding occasional tobacco users.
Occasional: Results from analyses excluding daily tobacco users.
Any: Results from analyses combining daily and occasional tobacco users.
κ: Cohen's kappa coefficient.
PPV: Positive predictive value
NPV: Negative predictive value

For peer review only

STROBE 2007 (v4) Statement—Checklist of items that should be included in reports of *cross-sectional studies*

Section/Topic	Item #	Recommendation	Reported on page #
**Title and abstract**	1	(*a*) Indicate the study's design with a commonly used term in the title or the abstract	1-2
		(*b*) Provide in the abstract an informative and balanced summary of what was done and what was found	2
**Introduction**			
Background/rationale	2	Explain the scientific background and rationale for the investigation being reported	4-5
Objectives	3	State specific objectives, including any prespecified hypotheses	5
**Methods**			
Study design	4	Present key elements of study design early in the paper	5-6
Setting	5	Describe the setting, locations, and relevant dates, including periods of recruitment, exposure, follow-up, and data collection	5-6
Participants	6	(*a*) Give the eligibility criteria, and the sources and methods of selection of participants	5-6
Variables	7	Clearly define all outcomes, exposures, predictors, potential confounders, and effect modifiers. Give diagnostic criteria, if applicable	7-8
Data sources/ measurement	8*	For each variable of interest, give sources of data and details of methods of assessment (measurement). Describe comparability of assessment methods if there is more than one group	7-8
Bias	9	Describe any efforts to address potential sources of bias	7-8
Study size	10	Explain how the study size was arrived at	5-6
Quantitative variables	11	Explain how quantitative variables were handled in the analyses. If applicable, describe which groupings were chosen and why	7-8
Statistical methods	12	(*a*) Describe all statistical methods, including those used to control for confounding	7-8
		(*b*) Describe any methods used to examine subgroups and interactions	7-8
		(*c*) Explain how missing data were addressed	8
		(*d*) If applicable, describe analytical methods taking account of sampling strategy	
		(*e*) Describe any sensitivity analyses	8
**Results**			

For peer review only

Participants	13*	(a) Report numbers of individuals at each stage of study—eg numbers potentially eligible, examined for eligibility, confirmed eligible, included in the study, completing follow-up, and analysed	5-6
		(b) Give reasons for non-participation at each stage	5-6
		(c) Consider use of a flow diagram	2 in supplement
Descriptive data	14*	(a) Give characteristics of study participants (eg demographic, clinical, social) and information on exposures and potential confounders	8-12
		(b) Indicate number of participants with missing data for each variable of interest	8 and Table 1
Outcome data	15*	Report numbers of outcome events or summary measures	12-13
Main results	16	(*a*) Give unadjusted estimates and, if applicable, confounder-adjusted estimates and their precision (eg, 95% confidence interval). Make clear which confounders were adjusted for and why they were included	12-14
		(*b*) Report category boundaries when continuous variables were categorized	12-14
		(*c*) If relevant, consider translating estimates of relative risk into absolute risk for a meaningful time period	
Other analyses	17	Report other analyses done—eg analyses of subgroups and interactions, and sensitivity analyses	13
**Discussion**			
Key results	18	Summarise key results with reference to study objectives	15
Limitations	19	Discuss limitations of the study, taking into account sources of potential bias or imprecision. Discuss both direction and magnitude of any potential bias	15, 16 & 18
Interpretation	20	Give a cautious overall interpretation of results considering objectives, limitations, multiplicity of analyses, results from similar studies, and other relevant evidence	15-18
Generalisability	21	Discuss the generalisability (external validity) of the study results	18
**Other information**			
Funding	22	Give the source of funding and the role of the funders for the present study and, if applicable, for the original study on which the present article is based	20

*Give information separately for cases and controls in case-control studies and, if applicable, for exposed and unexposed groups in cohort and cross-sectional studies.

Note: An Explanation and Elaboration article discusses each checklist item and gives methodological background and published examples of transparent reporting. The STROBE checklist is best used in conjunction with this article (freely available on the Web sites of PLoS Medicine at http://www.plosmedicine.org/, Annals of Internal Medicine at http://www.annals.org/, and Epidemiology at http://www.epidem.com/). Information on the STROBE Initiative is available at www.strobe-statement.org.

For peer review only
